# Quantitative Structure–Property Relationship (QSPR) Models for a Local Quantum Descriptor: Investigation of the 4- and 3-Substituted-Cinnamic Acid Esterification

**DOI:** 10.3390/molecules200917493

**Published:** 2015-09-22

**Authors:** Cláudio E. Rodrigues-Santos, Aurea Echevarria, Carlos M. R. Sant’Anna, Thiago B. Bitencourt, Maria G. Nascimento, Glauco F. Bauerfeldt

**Affiliations:** 1 Departamento de Química, Instituto de Ciências Exatas, Universidade Federal Rural do Rio de Janeiro-RJ, Seropédica 23890-900, Brazil; E-Mails: claudioers@ufrrj.br (C.E.R.-S.); bauerfeldt@ufrrj.br (A.E.); santanna@ufrrj.br (C.M.R.S.); 2 Departamento de Engenharia de Alimentos, Universidade Federal da Fronteira Sul, Laranjeiras do Sul-PR 85303-775, Brazil; E-Mail: thiago06br@yahoo.com.br; 3 Departamento de Química, Universidade Federal de Santa Catarina, Florianópolis-SC 88040-900, Brazil; E-Mail: maria.nascimento@ufsc.br

**Keywords:** *O*-protonation, cinnamic acid, FERMO, Fukui functions

## Abstract

In this work, the theoretical description of the 4- and 3-substituted-cinnamic acid esterification with different electron donating and electron withdrawing groups was performed at the B3LYP and M06-2X levels, as a two-step process: the *O*-protonation and the nucleophile attack by ethanol. In parallel, an experimental work devoted to the synthesis and characterization of the substituted-cinnamate esters has also been performed. In order to quantify the substituents effects, quantitative structure–property relationship (QSPR) models based on the atomic charges, Fukui functions and the Frontier Effective-for-Reaction Molecular Orbitals (FERMO) energies were investigated. In fact, the Fukui functions, ƒ^+^C and ƒ^−^O, indicated poor correlations for each individual step, and in contrast with the general literature, the *O*-protonation step is affected both by the FERMO energies and the *O*-charges of the carbonyl group. Since the process was shown to not be totally described by either charge- or frontier-orbitals, it is proposed to be frontier-charge-*miscere* controlled. Moreover, the observed trend for the experimental reaction yields suggests that the electron withdrawing groups favor the reaction and the same was observed for Step 2, which can thus be pointed out as the determining step.

## 1. Introduction

**Figure 1:**
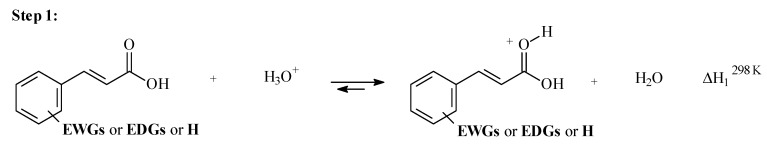

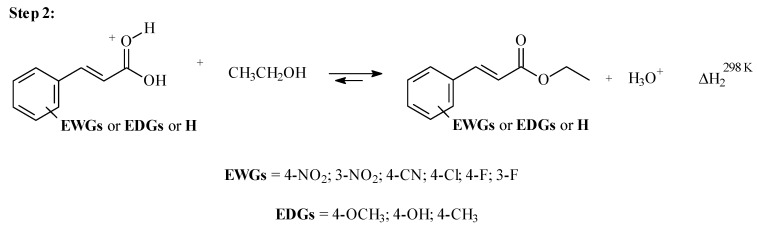
Steps for acid-catalyzed 4- or 3-X-cinnamic acid esterification.

Esters are compounds of natural or synthetic source, and they can be found in several materials, being extensively used in food industries, as constituents of some important flavor compounds. They are also found in honeys, flowers, fruits, and in fermented beverages, such as wine and beer. The cinnamates, ester derivatives of the cinnamic acid, besides acting as flavorings agents, can also be used as antioxidants, antifungal, anti-rheumatic, and even as inhibitions of the protein kinase C, a target for cancer treatment [1,2,3,4]. They are also widely used in the formulations of ultra radiation B, UVB (280–320 nm) and ultra radiation A, UVA (320–400 nm) absorbers [5]. Esters can be obtained by the Fisher esterification method, which is an acylation of alcohols by acid-catalyzed reaction with carboxylic acid. Recently, this method was described as one responsible reaction for formation of methyl formate in interstellar clouds [6]. The reactions are understood by their mechanistic aspects, and it demonstrates the selectivity and reactivity of the process. These can be expressed as charges, highest occupied molecular orbital (HOMO) and unoccupied molecular orbital (LUMO) energies, (HOMO-LUMO gap), Fukui function, and FERMOS (Frontier Effective-for-Reaction Molecular Orbitals) quantum descriptors [7]. Due to the importance of the cinnamates, and considering the interest both in studying the electronic effects arising from different substituents in cinnamoyl moiety [8,9] and in investigating the fundamental aspects that can elucidate the dynamic of chemical reactions [10,11], we report here theoretical-experimental results with the aim of describing a selection of quantum descriptors for the local hardness of carbonyl in the 4- and 3-substituted-cinnamic acid esterification being the substituents effects measured by quantitative structure–property relationship (QSPR). In order to assess such quantum descriptors, theoretical calculations have been performed at the density functional theory (DFT) level for a series of ethyl 4- and 3-substituted-cinnamates. The esterification process was theoretically described by two steps, the *O*-protonation, Step 1, and the nucleophile attack by ethanol, Step 2, as suggested [12]. A general scheme for the two steps is shown in Figure 1. Moreover, the synthesis of the same compounds was conducted in order to provide experimental parameters for comparison with the theoretical results.

### Theoretical Background and Computational Details

The effects of the substituents in a chemical reaction have always been of great interest of chemists. A quantitative treatment of such effects has been described by the pioneering work of Hammett in 1937. The author proposed a linear free-energy relationship, represented by the equation below [12,13,14].

(1) $$\sigma_{X}=\text{log}K_{X}-\text{log}K_{H}$$

In Equation (1), *K_X_* is the ionization equilibrium constant for a substituted benzoic acid and K_H_ is the ionization equilibrium constant for benzoic acid. The Hammett substituent constants values have been employed for the understanding of several organic reactions and their related mechanisms [14,15,16]. In fact, the understanding of chemical reactions mechanisms from the microscopic point of view has been a great challenge for chemistry researchers. In this context, Lewis proposed that most of the chemical reactions can be described as an acid–base process, being the acids, electron-pair acceptors and bases the electron-pair donors [17]. In 1963, Pearson introduced the hard and soft acid–base (HSAB) concept, which conveniently divided acids and bases into the following categories: hard, soft and borderline. In this approach, hard acids preferentially react with hard bases, whereas soft acids preferentially react with soft bases [18]. Although the HSAB concept has received great attention in the chemical community, explaining not only inorganic but also organic reactions, the lack of a quantitative description for the theory resulted in a lot of criticism [19,20,21]. In 1968, Klopman [22] affirmed that if the |*E*_HOMO_ − *E*_LUMO_| ~ 0, the interaction between orbitals becomes predominant, being this reaction referred as a frontier-controlled process, whereas if |*E*_HOMO_ − *E*_LUMO_| >> 0, the electron is transferred, and this reaction is referred as a charge-controlled process. In 1983, Parr demonstrated that every chemical system can be associated to the so-called electronic-chemical potential, defining also the chemical hardness according to the density functional theory (DFT), thus the HSAB concept reemerged. Using the molecular orbitals (MO) energies, the larger the energy gap between *E*_LUMO_ − *E*_HOMO_, the harder is the species, being the hardness defined from Equation (2) [23,24].

(2) $$\text{η}=\frac{1}{2}\left(E_{HOMO}-E_{LUMO}\right)$$

In 2007, Anderson and coworkers, working out all these concepts, suggested that some reactions are in an interface, neither totally charge- nor totally frontier-orbital-controlled [25]. Even with the increasing computational capabilities and all the effort devoted for the comprehension of the chemical reactions, the HOMO-LUMO approach cannot explain certain reactions, mainly those involving ambidentate molecules. The local hardness and softness concept can also be represented by the Fukui Functions (ƒ(*r*)), which is formally defined as the partial derivative of the chemical potential, μ, with respect to an external potential, ν(*r*), at a constant number of electrons (*N*) [26]:

(3) $$f(r)={\left(\frac{\partial \text{μ}}{\partial \text{ν}(r)}\right)}_{N}={\left(\frac{\partial \rho (r)}{\partial N}\right)}_{\text{ν}(r)}$$

On the basis of the discontinuity of the ƒ(*r*) *versus* *N* curve, three types of Fukui functions can be defined: the $f_{k}^{+}$ and $f_{k}^{-}$ functions, which account for nucleophilic and electrophilic attacks, respectively:

(4) $$f_{k}^{+}=\left[q_{k}\left(N+1\right)-q_{k}\left(N\right)\right]$$

(5) $$f_{k}^{-}=\left[q_{k}\left(N\right)-q_{k}\left(N-1\right)\right]$$

and the $f_{k}^{0}$ function:

(6) $$f_{k}^{0}=\frac{1}{2}\left[q_{k}\left(N+1\right)-q_{k}\left(N-1\right)\right]$$

which accounts for the homolytic attacks. In these equations, *q_k_* is the gross charge of atom k in the molecule and *N* is the number of electrons. It must be highlighted that only Equations (4) and (5) are of interest in this work. Within these definitions, several reactions could be explained [27]. Some researchers have considered that the Fukui function represents a good measurement of the chemical hardness [28], and others have demonstrated that the protonation sites can be estimated from the investigation of this function [29,30]. Melin *et al.* demonstrated that for hard-hard interaction, the atomic charges were the most appropriate descriptor for the protonation reaction of hydroxylamine and some amino acids, performing even better than the Fukui function [31]. An alternative way to describe local hardness was proposed by Silva *et al.* who introduced the Frontier Effective-for-Reaction Molecular Orbital (FERMO) concept [32], that despite some contestations [33,34], has been showed to perform satisfactorily for the global understanding of ambidentate species selectivity such as SCN^−^, NO_2_^−^, CH_3_OCH_2_^−^ and *N*,*N*-dimethylsulfoxide (DMSO). Within the FERMO concept, the local hardness is redefined, as [35]:

(7) $$\text{η}\prime =\frac{1}{2}\left(E_{FERMO}-E_{LUMO}\right)$$

In Equation (7), the FERMO corresponds to a HOMO-_X_, a given occupied molecular orbital showing the greatest contribution to compose the reactive center [36]. All these cited reactivity indexes can bring thermodynamic as much as kinetics considerations [37].

## 2. Methodology

### 2.1. Synthesis and Characterization

^1^H-NMR chemical shifts were determined at room temperature on a Bruker AC (200 or 400 MHz) spectrometer in CDCl_3_ or DMSO-*d*_6_ with TMS as internal standard. The ^13^C spectra were determined on a Varian EM 360L (100 MHz) spectrometer. Instrumental conditions were such as follow: relax delay 1 s, pulse 45°, and data process FT size 65536, spectral width 6398.0 Hz. The reaction progress was measured by gas chromatography on a GC-Shimadzu-14B equipped with a medium polar column (Shimadzu CBP5-25m) using H_2_ as the carrier gas, with a flame ionization detector set at 280 °C, an injector set at 270 °C and a column set to a temperature range from 50 to 250 °C (10 °C/min). Infrared spectra were recorded on a Perkin Elmer FT 16-PC and the most intense or representative bands are reported in cm^−1^. Melting points were determined on a Microquímica model APF 301 apparatus and are uncorrected.

A series of 4- or 3-substituted ethyl-cinnamates was prepared by conventional method [38], using 2 mmol of *para* and *meta* substituted cinnamic acids refluxed in absolute ethanol (15 mL) and sulfuric acid (0.1 mL) for 30 h. The ester formation was monitored by GC and TLC (hexane:acetate 9:1 as eluent). The products were isolated after washing with aqueous sodium bicarbonate solution and solvent extraction with dichloromethane. After work up and solvent evaporation, most of the pure esters were obtained as an oil or a solid. All compounds were characterized by ^1^H and ^13^C-NMR spectroscopy, in agreement with the literature [39,40,41]. The spectroscopic and characterization information of all compounds is given as Electronic Supplementary Information.

### 2.2. Computational Methods

In order to assess the local quantum descriptors described above, quantum mechanical calculations have been performed for the same set of ethyl 4- or 3-substituted-cinnamates with different electron donating (EDGs) and electron withdrawing (EWGs) groups. Geometry optimizations have been performed at the B3LYP [42] level adopting the 6-31+G(d,p) basis set. Scan calculations over some dihedral angles have been conducted in order to guarantee that these geometries correspond to the most stable conformers. The characterization as a minimum energy geometry has also been done by calculating and inspection of the vibrational frequencies, at the same level. The B3LYP functional is probably the most popular and worldwide used functional. Its application for assessing reaction mechanisms, however, has been discussed and the M06-2X functional, a meta exchange-correlation functional proposed by Truhlar and coworkers [43], has been proven much more reliable for investigations on reaction mechanisms. Therefore, additional M06-2X/6-31+G(d,p) calculations for geometry optimizations and vibrational frequencies were performed. It must be noticed that, due to the size of the systems, calculations at a more robust theoretical level, as CCSD(T), are unfeasible.

The orbital eigenvalues have been obtained through single point calculations over the B3LYP/6-31+G(d,p) optimized geometries, at the restricted hartree-fock level adopting the 6-311++G(2d,2p) basis set. The more flexible basis set was adopted in an attempt to obtain improved orbital (HOMO, LUMO and the FERMO, HOMO-x_1_ and HOMO-x_2_) energy values. Local hardness parameters for the 4- or 3-X-cinnamic acid were calculated according to da Silva *et al.* [32,35]:

(8) $${\text{η}}^{1}=\frac{1}{2}\left(E_{HOMO-X_{1}}-E_{LUMO}\right)$$

(9) $${\text{η}}^{2}=\frac{1}{2}\left(E_{HOMO-X_{2}}-E_{LUMO}\right)$$

In a similar way, the local hardness parameters for the protonated 4- or 3-X-cinnamic acid were calculated as:

(10) $${\text{η}}^{3}=\frac{1}{2}\left(E_{HOMO-X_{1}}-E_{LUMO}\right)$$

(11) $${\text{η}}^{4}=\frac{1}{2}\left(E_{HOMO-X_{2}}-E_{LUMO}\right)$$

The global hardness parameters were also calculated.

In order to evaluate the ƒ^−^O and ƒ^+^C Fukui functions, single point calculations have been performed at B3LYP and M06-2X levels adopting the 6-311++G(2d,2p) basis set, over the optimized geometry, located at corresponding theoretical level along with the 6-31+G(d,p) basis set. In order to measure the charges for the (N + 1) and (N − 1) species, similar single point calculations were performed over the previously optimized geometries, changing the charge number. The atomic charges were obtained from Natural Population Analysis phase of NBO calculations. Using the atomic charges over the oxygen and carbon atoms connected to the carbonyl group, Fukui functions were evaluated according to Equations (4) and (5).

Standard thermochemical properties have been obtained by conventional relations [44], adopting the ideal gas, rigid rotor and harmonic oscillator models. All theoretical calculations have been performed for the isolated systems, using Gaussian program [45].

### 2.3. Statistics Analysis

The models were obtained by linear regression analyses using the Build QSAR program to determine the parameters. Throughout this paper, n is number of data points, r is the correlation coefficient, Sd is the standard error, and F is Fisher value for the statistical significance [46].

## 3. Results and Discussion

A series of 10 ethyl 4- or 3-X-cinnamates were synthesized from 4- or 3-X-cinnamic acid with ethanol, in accordance with the literature [10], where X = 4-OCH_3_, 4-OH, 4-CH_3_ electron donor groups (EDGs), 3-F, 3-NO_2_, 4-Cl, 4-F, 4-CN, 4-NO_2_ electron withdrawing (EWGs) groups and X = H. The reactions were conducted under reflux for 30 h using sulfuric acid as catalyst, to furnish the ethyl 4- or 3-X-cinnamates in good yields (45%–80%), and that the compounds with EWGs presented the best results. The compounds were obtained in *trans* geometry, being confirmed by the values of the coupling constant (*J*) of the olefinic hydrogens in the range of 15.0–16.5 Hz [47].

### 3.1. Theoretical Calculations

**Figure 2:**
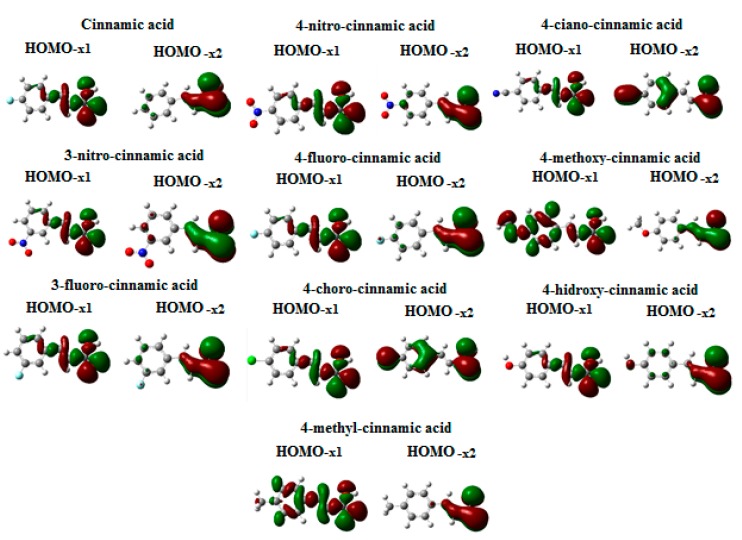
Surface plots for HOMO-x_1_ and HOMO-x_2_ molecular orbitals for the compounds.

For a complete theoretical description of the esterification reaction, the molecular geometries for reactants, products and intermediates were optimized and, as expected, all vibrational frequencies were determined as real values, characterizing all the geometries as minima. The resulting geometries and other molecular properties are given as Electronic Supplementary Information. Moreover, as stated above, potential energy curves were calculated in order to guarantee that these geometries correspond to the most stable conformation of each molecule and the results obtained for the cinnamic acid, protonated cinnamic acid and ethyl cinnamate are also given as Electronic Supplementary Information. Only those obtained for cinnamic acid, protonated cinnamic acid and ethyl cinnamate were reported, since similar behavior was found for the compounds. As it can be noted from the potential curves, cinnamic acids and protonated cinnamic acids are most stable at the planar conformation. Concerning the ethyl cinnamates, B3LYP predicts stable conformers showing the carbon atoms of the ethyl group at the same plane as the aromatic ring. This situation is avoided at the M06-2X level, and the terminal CH_3_ group is found with a dihedral angle (CCOC) of nearly 90°. Except for the CH_3_ group (and H atoms at ethyl CH_2_ group), all atoms lie at the same plane. The theoretical calculations were performed considering isolated systems, although the reactions were experimentally conducted in the presence of the solvent. Nevertheless, it has been shown that negligible contribution is observed by performing calculations considering a solvent model, in comparison with the gas phase results [48]. The standard enthalpy differences were obtained for each step (ΔH_1_ and ΔH_2_, respectively). The Frontier Effective-for-Reaction Molecular Orbitals (FERMOs), shown in Figure 2, have been chosen as the molecular orbitals with the major contributions from the electron density on the carbonyl group atoms, thus, to the reactive center.

The quantum descriptors *E*_HOMO-x1_, *E*_HOMO-x2_, *E*_HOMO_ and *E*_LUMO_, corresponding to FERMOs, HOMO and LUMO energies, respectively, obtained for the 4- and 3-substituted-cinnamic acids and their protonated analogues and hardness parameters (η, η^1^, η^2^, η^3^ and η^4^) are reported in Table 1 and Table 2. In these Tables, Hammett substituent constants, standard reaction enthalpy differences, atomic charges and Fukui functions are also shown. Both B3LYP and M06-2X results are given.

### 3.2. Step 1: O-Protonation for Acid-Catalyzed 4- or 3-X-Cinnamic Acid Esterification

**Figure 3:**
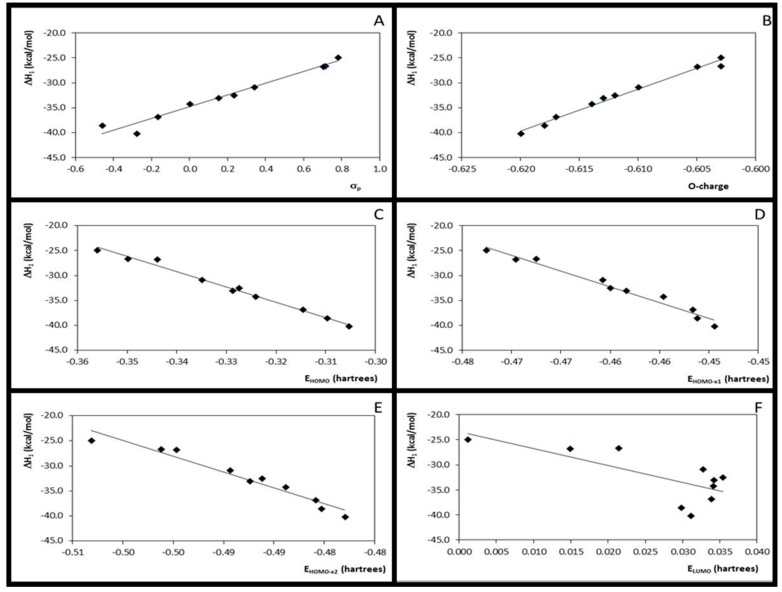
Plots for the correlation between enthalpies (ΔH_1_) values and Hammett substituent constants (**A**); *O*-charges (**B**) and HOMO, HOMO-x_1_, HOMO-x_2_ and LUMO energies (**C**–**F**, respectively).

The substituent effects in the *O*-protonation step were observed by the correlations found between Hammett constants (σ_p_) and *O*-charge (r^2^ = 0.96, F = 197, for the B3LYP data and r^2^ = 0.96, F = 178, for the M06-2X data) and by correlation between σ_p_ and ΔH_1_ (r^2^ = 0.96, F = 184, for the B3LYP data and r^2^ = 0.97, F = 231, for the M06-2X data). The atomic charges were, in particular, good local descriptors also showing good correlation with the ΔH_1_ values: r^2^ = 0.99 and 0.98 (B3LYP and M06-2X, with F values 775 and 512, respectively), suggesting that the first step is favored by the EDGs. These data are still corroborated by the good correlation found between σ_p_ and ΔH_1_. In fact, it may be expected that the EDGs increase the *O*-charge, favoring this reaction step. Despite some authors have showed the Fukui functions are good descriptors for the protonation sites [29,30], the ƒ^−^O and the local hardness showed poor correlation with the *O*-protonation enthalpy variation (r^2^ = 0.45, F = 6.53 and r^2^ = 0.61, F = 12.69, for the B3LYP and M06-2X results, respectively). These results are corroborated with the conclusions of Melin *et al.* [31]. Moreover, no QSPR-Models could be established for the local hardness, η^1^ and η^2^. The FERMOs energies *E*_HOMO-x1_ and *E*_HOMO-x2_, as well as *E*_HOMO_, however, well correlate with the first step enthalpy (ΔH_1_). The statistical correlation parameters obtained for ΔH_1_ *versus* *E*_HOMO_ were: r^2^ = 0.99 and F = 967 (B3LYP) and r^2^ = 0.99 and F = 675 (M06-2X). For ΔH_1_ *versus* *E*_HOMO-x1_, the following statistical correlation parameters have been observed: r^2^ = 0.97 and F = 223 (B3LYP) and r^2^ = 0.97 and F = 269 (M06-2X), while for ΔH_1_ *versus E*_HOMO-x2_, r^2^ = 0.95 and F = 153 (B3LYP) and r^2^ = 0.95 and F = 141 (M06-2X) were found. Weak correlation was observed for ΔH_1_ *versus E*_LUMO_. Statistical parameters for the several possible correlations are summarized in Table 3. These relations, obtained at the M06-2X level, can be observed in Figure 3. B3LYP data are here omitted, since no significant changes from the M06-2X data were observed.

**Table 1:** Calculated properties for the 4- or 3-X-cinnamic acids: Hammett substituent constants (σ_p_), molecular orbital energies (in hartrees), hardness parameters (η, η^1^ and η^2^, in hartrees), standard enthalpy variation (first step, in kcal/mol), atomic charges and ƒ^−^O Fukui function. B3LYP and M06-2X results are both given for comparison.

	X	σ_p_	*E*_HOMO-x2_	*E*_HOMO-x1_	*E*_HOMO_	*E*_LUMO_	η	η^1^	η^2^	B3LYP			M06-2X		
										ΔH_1_^298K^	*O*-Charge	ƒ^−^O	ΔH_1_^298K^	*O*-Charge	ƒ^−^O
			(Hartrees)	(Hartrees)	(Hartrees)	(Hartrees)	(Hartrees)	(Hartrees)	(Hartrees)	(kcal/mol)			(kcal/mol)		
**1**	H	0	−0.4839	−0.4547	−0.3241	0.034	−0.1791	−0.2444	−0.2590	−39.35	−0.609	−0.114	−34.23	−0.614	−0.104
**2**	4-NO_2_	0.78	−0.5032	−0.4726	−0.3559	0.001	−0.1785	−0.2369	−0.2522	−29.71	−0.596	−0.114	−24.98	−0.603	−0.107
**3**	3-NO_2_	0.71	−0.4963	−0.4676	−0.3498	0.021	−0.1856	−0.2445	−0.2589	−31.51	−0.597	−0.116	−26.68	−0.603	−0.107
**4**	4-F	0.15	−0.4874	−0.4584	−0.3288	0.034	−0.1815	−0.2463	−0.2608	−38.08	−0.608	−0.112	−33.04	−0.613	−0.103
**5**	3-F	0.34	−0.4894	−0.4609	−0.3349	0.033	−0.1838	−0.2468	−0.2611	−36.00	−0.604	−0.112	−30.87	−0.610	−0.103
**6**	4-Cl	0.23	−0.4862	−0.4600	−0.3274	0.035	−0.1814	−0.2477	−0.2608	−37.97	−0.607	−0.103	−32.47	−0.612	−0.095
**7**	4-CN	0.70	−0.4948	−0.4697	−0.3440	0.015	−0.1795	−0.2423	−0.2549	−31.92	−0.599	−0.105	−26.77	−0.605	−0.099
**8**	4-OCH_3_	−0.28	−0.4780	−0.4495	−0.3053	0.031	−0.1682	−0.2403	−0.2546	−46.04	−0.616	−0.098	−40.24	−0.620	−0.089
**9**	4-OH	−0.46	−0.4804	−0.4512	−0.3096	0.030	−0.1697	−0.2405	−0.2551	−43.97	−0.614	−0.103	−38.60	−0.618	−0.093
**10**	4-CH_3_	−0.17	−0.4809	−0.4517	−0.3145	0.034	−0.1742	−0.2428	−0.2574	−42.33	−0.612	−0.106	−36.88	−0.617	−0.097

**Table 2:** Calculated properties for the protonated 4- or 3-X-cinnamic acids: Hammett substituent constants (σ_p_), molecular orbital energies (in hartrees), hardness parameters (η, η^3^ and η^4^, in hartrees), standard enthalpy variation (second step, in kcal/mol), atomic charges and ƒ^+^C Fukui function. B3LYP and M06-2X results are both given for comparison.

	X	σ_p_	*E*_HOMO-x2_	*E*_HOMO-x1_	*E*_HOMO_	*E*_LUMO_	η	η^3^	η^4^	B3LYP			M06-2X		
										ΔH_2_^298K^	*C*-Charge	ƒ^+^C	ΔH_2_^298K^	*C*-Charge	ƒ^+^C
			(Hartrees)	(Hartrees)	(Hartrees)	(Hartrees)	(Hartrees)	(Hartrees)	(Hartrees)	(kcal/mol)			(kcal/mol)		
**1**	H	0	−0.8494	−0.7366	−0.4652	−0.133	−0.1660	−0.3018	−0.3582	37.50	0.809	−0.165	31.09	0.854	−0.187
**2**	4-NO_2_	0.78	−0.8703	−0.7555	−0.4938	−0.158	−0.1678	−0.2987	−0.3560	27.38	0.822	−0.153	21.51	0.869	−0.179
**3**	3-NO_2_	0.71	−0.8658	−0.7513	−0.4888	−0.149	−0.1701	−0.3013	−0.3586	29.30	0.821	−0.164	23.35	0.868	−0.188
**4**	4-F	0.15	−0.8525	−0.7381	−0.4682	−0.135	−0.1666	−0.3016	−0.3588	36.18	0.806	−0.163	29.90	0.851	−0.185
**5**	3-F	0.34	−0.8575	−0.7439	−0.4719	−0.142	−0.1651	−0.3011	−0.3579	34.03	0.814	−0.165	27.67	0.860	−0.188
**6**	4-Cl	0.23	−0.8512	−0.7380	−0.4590	−0.138	−0.1607	−0.3002	−0.3568	35.98	0.805	−0.157	29.26	0.851	−0.179
**7**	4-CN	0.70	−0.8647	−0.7506	−0.4753	−0.154	−0.1607	−0.2984	−0.3554	29.70	0.817	−0.155	23.36	0.864	−0.178
**8**	4-OCH_3_	−0.28	−0.8329	−0.7161	−0.4391	−0.120	−0.1596	−0.2981	−0.3565	44.38	0.785	−0.152	37.31	0.829	−0.171
**9**	4-OH	−0.46	−0.8386	−0.7227	−0.4467	−0.124	−0.1614	−0.2994	−0.3574	42.28	0.790	−0.155	35.68	0.835	−0.176
**10**	4-CH_3_	−0.17	−0.8425	−0.7283	−0.4517	−0.127	−0.1623	−0.3006	−0.3577	40.53	0.800	−0.159	33.80	0.846	−0.181

**Table 3:** Correlations analysis between local properties and enthalpy variation for 4- or 3-X-cinnamic acid protonation (Step 1), obtained from B3LYP and M06-2X results.

Entry	Correlation	Statistic Parameters, from B3LYP Data						Statistic Parameters, from M06-2X Data					
		^a^ n	^b^ r^2^	^c^ Sd	^d^ F	^e^ a	^f^ b	^a^ n	^b^ r^2^	^c^ Sd	^d^ F	^e^ a	^f^ b
**1**	ΔH_1_ × σ_p_	10	0.96	1.190	183.76	12.332	−40.156	10	0.97	1.012	231.80	11.773	−34.831
**2**	ΔH_1_ × E_HOMO_	10	0.99	0.528	967.21	−322.772	−144.019	10	0.99	0.600	674.59	−306.266	−133.368
**3**	ΔH_1_ × E_HOMO-x1_	10	0.97	1.085	222.72	−663.279	−342.544	10	0.97	0.942	268.58	−632.353	−323.116
**4**	ΔH_1_ × E_HOMO-x2_	10	0.95	1.299	152.98	−669.555	−364.463	10	0.95	1.282	141.37	−635.148	−342.458
**5**	ΔH_1_ × E_LUMO_	10	0.53	4.003	8.96	−357.354	−28.077	10	0.53	3.806	8.95	−339.609	−23.341
**6**	ΔH_1_ × η^1^	10	0.00	5.827	0.003	30.301	−30.319	10	0.00	5.540	0.002	22.962	−26.891
**7**	ΔH_1_ × η^2^	10	0.00	5.821	0.021	90.116	−14.488	10	0.00	5.532	0.023	89.398	−9.459
**8**	ΔH_1_ × *O*-charge	10	0.99	0.610	722.05	774.924	432.069	10	0.98	0.687	512.40	840.187	481.298
**9**	*O*-charge × σ	10	0.96	0.001	197.14	0.016	−0.609	10	0.96	0.001	177.59	0.014	−0.614
**10**	ΔH_1_ × ƒ^−^O	10	0.45	4.324	6.53	−608.324	−103.572	10	0.61	3.445	12.69	−669.405	−99.216

^a^ number of data points; ^b^ square correlation coefficient; ^c^ standard deviation; ^d^ F test for significance of correlation; ^e^ slope; ^f^ intercept.

These results revealed that for Step 1, the frontier orbitals are as important as the charges. The importance of the frontier orbitals for the reaction process control has been revealed by the QSPR-models obtained for *E*_HOMO_, *E*_HOMO-x1_ and *E*_HOMO-x2_, whilst the good correlations with the reaction thermochemical property, ΔH_1_, suggested that the charges are also good reaction descriptors. In this way, the first step cannot be considered neither totally charge- nor totally frontier-orbital-controlled, and in the lack of an appropriate term, we refer to Step 1 as a frontier-charge-*miscere* controlled reaction.

### 3.3. Step 2: The Nucleophilic Attack by Ethanol for Acid-Catalysed 4- or 3-X-Cinnamic Acid Esterification

**Figure 4:**
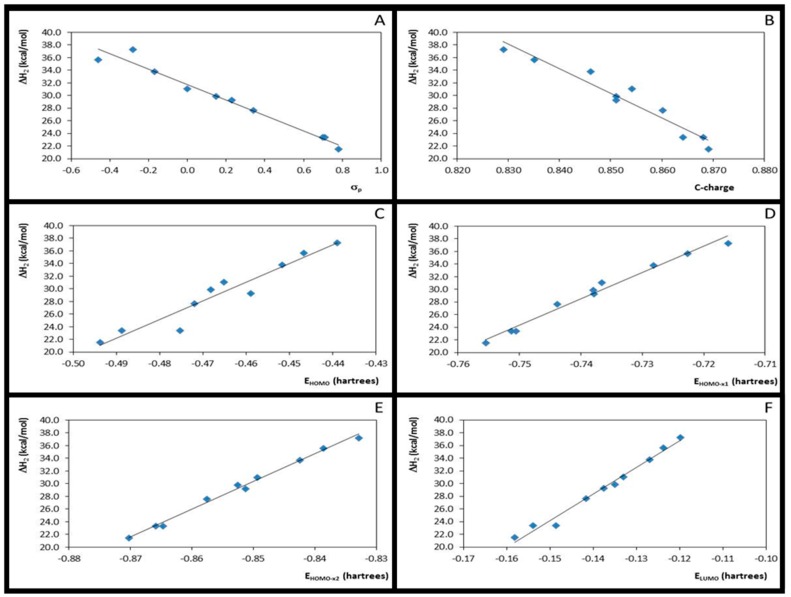
Plots for the correlation between enthalpies (ΔH_2_) values and Hammett substituent constant (**A**); *C*-charges (**B**); and HOMO, HOMO-x_1_, HOMO-x_2_ and LUMO energies (**C**–**F**, respectively).

The substituents effects in the Step 2 were also assessed by the possible correlation among the quantum descriptors. It was found that the Hammett constants (σ_p_) correlates well with both *C*-charge (r^2^ = 0.87, F = 55, B3LYP and r^2^ = 0.88, F = 59, M06-2X) and ΔH_2_ (r^2^ = 0.96, F = 189, B3LYP and r^2^ = 0.97, F = 237, M06-2X). It was also observed, as expected, that the EWGs increase the *C*-charge, consequently favoring this step, in the contrast to the Step 1. In fact, the *C*-charges were proved to be good quantum descriptor for the nucleophilic step, showing good correlation with the ΔH_2_ values (r^2^ = 0.93, F = 110, B3LYP and r^2^ = 0.94, F = 115, M06-2X). Strong correlations were observed between the occupied molecular orbital energies and ΔH_2_ values, as observed from the statistical correlation parameters: r^2^ = 0.94, F = 123, r^2^ = 0.98, F = 491 and r^2^ = 1.00, F = 1876 for ΔH_1_ *versus* *E*_HOMO_, ΔH_1_ *versus* *E*_HOMO-x1_ and ΔH_1_ *versus* *E*_HOMO-x2_, respectively (B3LYP data), and r^2^ = 0.92, F = 98, r^2^ = 0.98, F = 419 and r^2^ = 0.99, F = 1035 for ΔH_1_ *versus* *E*_HOMO_, ΔH_1_ *versus* *E*_HOMO-x1_ and ΔH_1_ *versus* *E*_HOMO-x2_, respectively (M06-2X data). Moreover, the lowest unoccupied molecular orbital energy, ELUMO, strongly correlates with ΔH_2_ values, (r^2^ = 0.98, F = 404, B3LYP and r^2^ = 0.98, F = 504, M06-2X). Neither the local hardness parameters (η^3^ and η^4^) nor the Fukui function (ƒ^+^C) were shown to be good descriptors for this reaction step. Statistical parameters are summarized in Table 4 and graph correlations, obtained at the M06-2X level, can be observed in Figure 4. In Step 2, as also noted in Step 1, strong correlations were between the ΔH values and *C*-charges and between the ΔH values FERMO energies, but not with Fukui Function or hardness parameters, suggesting that this step is also governed by electrostatic interactions, as well as by frontier orbitals. Therefore, the whole process should be better described as a frontier-charge-*miscere* controlled reaction.

### 3.4. Global Reaction: Theoretical and Experimental Data Compared

As no significant differences between B3LYP and M06-2x results for Steps 1 and 2 were observed, only correlations from B3LYP data will be discussed in this topic. From the data in Table 5, a good correlation between yield (%) and Hammett constants can be observed (entry 1, r^2^ = 0.89, F = 64.52), as well as between Hammett constants and global enthalpy (entry 3, r^2^ = 0.96, F = 189.96), demonstrating that the yield is favored by withdrawing groups. As expected, correlation between yield and global enthalpy (entry 2, r^2^ = 0.88, F = 53.91) can be noted with one outlier, 4-hydroxy-cinnamic acid (**9**). Same tendency (negative slope) was observed between the correlation of the second step enthalpy and yield (entry 5, r^2^ = 0.91, F = 74.68), however, the opposite (positive slope) was observed for correlation between yield and first step enthalpy (entry 4, r^2^ = 0.91, F = 74.59). There is a strong correlation between *E*_LUMO_ and yield (entry 9, r^2^ = 0.94, F = 112.08), which is, interestingly, a more expressive correlation than that observed for the *C*-charge (entry 11, r^2^ = 0.80, F = 4.816). Since the correlation with the *E*_LUMO_ is expected for a nucleophilic substitution, the second step is suggested as the most important for the whole process. Once again, as occurred in Steps 1 and 2, poor correlations utilizing the Fukui function (ƒ^−^*O*) and hardness parameters (η, η^1^, η^2^, η^3^, and, η^4^) were observed (entry 12–18), however, good correlations among orbitals energies (*E*_HOMO-x2_, *E*_HOMO-x1_ and *E*_HOMO_) can be seen in Table 5 (entry 6, r^2^ = 0.88, F = 51.93, entry 7, r^2^ = 0.91, F = 76.06, entry 8, r^2^ = 0.90, F = 64.10, respectively).

**Table 4:** Correlations analysis between local properties and enthalpy variation for the protonated 4- or 3-X-cinnamic acid reactions (Step 2), obtained from B3LYP and M06-2X results.

Entry	Correlation	Statistic Parameters, from B3LYP Data						Statistic Parameters, from M06-2X Data					
		^a^ n	^b^ r^2^	^c^ Sd	^d^ F	^e^ a	^f^ b	^a^ n	^b^ r^2^	^c^ Sd	^d^ F	^e^ a	^f^ b
**1**	ΔH_2_ × σ_p_	10	0.96	1.224	188.79	−12.848	38.295	10	0.97	1.036	236.73	−12.179	31.728
**2**	ΔH_2_ × E_HOMO_	10	0.94	1.502	122.67	315.800	182.878	10	0.92	1.575	97.82	295.839	167.144
**3**	ΔH_2_ × E_HOMO-x1_	10	0.98	0.768	491.36	440.946	361.197	10	0.98	0.784	418.70	415.698	336.128
**4**	ΔH_2_ × E_HOMO-x2_	10	1.00	0.395	1876.39	465.138	432.281	10	0.99	0.502	1034.83	438.358	403.016
**5**	ΔH_2_ × E_LUMO_	10	0.98	0.846	404.12	442.778	96.782	10	0.98	0.716	504.41	418.814	87.044
**6**	ΔH_2_ × η^3^	10	0.00	6.065	0.010	143.048	78.656	10	0.00	5.728	0.003	76.029	52.109
**7**	ΔH_2_ × η^4^	10	0.04	5.958	0.299	−975.353	−312.797	10	0.04	5.601	0.370	−1020.168	−335.244
**8**	ΔH_2_ × *C*-charge	10	0.93	1.580	110.04	−442.790	393.012	10	0.94	1.460	115.22	−390.752	362.487
**9**	*C*-charge × σ	10	0.87	0.005	55.32	0.027	0.802	10	0.88	0.005	58.93	0.029	0.847
**10**	ΔH_2_ × ƒ^+^C	10	0.03	5.987	0.22	184.019	64.947	10	0.18	5.179	1.79	405.806	102.824

^a^ number of data points; ^b^ square correlation coefficient; ^c^ standard deviation; ^d^ F test for significance of correlation; ^e^ slope; ^f^ intercept.

**Table 5:** Correlations analysis among global properties and enthalpies variations or local properties for the protonated 4- or 3-X-cinnamic acid (PCA) or 4- or 3-X-cinnamic acid (CA), obtained from B3LYP.

Entry	Correlation	Statistic Parameters, from B3LYP Data					
		^a^ n	^b^ r^2^	^c^ Sd	^d^ F	^e^ a	^f^ b
**1**	Yield × σ_p_	10	0.89	3.659	64.52	22.459	61.408
**2**	Yield × ΔH_r_	9	0.88	2.823	53.91	−33.197	2.085
**3**	σ_p_ × ΔH_r_	10	0.96	9.307	189.96	−186.546	−346.005
**4**	Yield × ΔH_1_	9	0.91	2.439	74.59	0.014	119.852
**5**	Yield × ΔH_2_	9	0.91	2.438	74.68	−0.013	115.165
**6**	Yield × *E*_HOMO-x2_ (CA) ^g^	9	0.88	2.870	51.93	−0.914	−378.864
**7**	Yield × *E*_HOMO-x1_ (CA) ^g^	9	0.91	2.418	76.06	−0.092	−358.353
**8**	Yield × *E*_HOMO_ (CA) ^g^	9	0.90	2.613	64.10	−0.045	−81.343
**9**	Yield × *E*_LUMO_ (PCA) ^h^	9	0.94	2.019	112.08	−0.604	−16.153
**10**	Yield × *O*-charge (CA) ^g^	9	0.87	2.900	50.73	1.059	709.663
**11**	Yield × *C*-charge (PCA) ^h^	10	0.80	4.816	33.86	0.748	−538.252
**12**	Yield × ƒ^−^*O* (CA) ^g^	10	0.32	9.030	3.90	−0.982	−40.491
**13**	Yield × η (CA) ^g^	10	0.60	6.979	11.93	−1396.507	−182.887
**14**	Yield × η^1^ (CA) ^g^	10	0.01	10.932	0.123	−379.821	−26.491
**15**	Yield × η^2^ (CA) ^g^	10	0.01	10.978	0.05	−275.984	−5.160
**16**	Yield × η (PCA) ^h^	10	0.24	9.595	2.54	−1431.193	−168.858
**17**	Yield × η^3^ (PCA) ^h^	10	0.00	10.997	0.03	−449.46	−68.993
**18**	Yield × η^4^ (PCA) ^h^	10	0.05	10.726	0.43	2100.165	−816.352

^a^ number of data points; ^b^ square correlation coefficient; ^c^ standard deviation; ^d^ F test for significance of correlation; ^e^ slope; ^f^ intercept; ^g^ cinnamic acid; ^h^ protonated cinnamic acid.

## 4. Conclusions

The effect of different EWGs and EDGs over the carbonyl was investigated by establishing QSPR-models for each reaction step in the esterification mechanism. It was possible to observe that the EDGs favored the first step (*O*-protonation), whereas the EWGs contributed to the second step (the ethanol attack). The experimental results suggest that the greatest yields were obtained with EWGs; these yields represent the global process, Step 2, following same trend as the experimental results, suggesting that this step is fundamental for the global esterification reaction. The global hardness could not be pointed out as a good descriptor for the 4- and 3-substituted-cinnamic acid esterification. From the QSPR-models, the *O*-protonation step for the 4- and 3-substituted-cinnamic acids cannot be considered to be controlled, neither by charges nor by the frontier-orbitals. Thus, a frontier-charge-*miscere* controlled process is suggested. In this context, the *O*-protonation reaction depends on the Lewis base and not only hydronium ion.

## Supplementary Materials

Supplementary materials can be accessed at: http://www.mdpi.com/1420-3049/20/09/17493/s1.
