# Supplementary material for: Quantitative Structure–Property Relationship (QSPR) Models for a Local Quantum Descriptor: Investigation of the 4- and 3-Substituted-Cinnamic Acid Esterification
Source: Molecules. 2015 Sep 22;20(9):17493–510. doi: 10.3390/molecules200917493 (PMC6331874; doi:10.3390/molecules200917493)
Supplement: Supplementary file 1 [file molecules-20-17493-s001.pdf]

Supplementary Data

1. Spectroscopic Data

Ethyl 3- and 4-X-cinnamates characterization 2

2. Molecular Properties (Coordinates, in Angstroms, vibrational Frequencies, in cm^{-1}, and Electronic Energy, in Hartreess) at the B3LYP/6-31+G(d,p) Level

Table S1. Cinnamic Acid 3
Table S2. Cinnamic Acid, Protonated 4
Table S3. Ethyl Cinnamate 5
Table S4. 4-Nitro-Cinnamic Acid 6
Table S5. 4-Nitro-Cinnamic Acid, Protonated 7
Table S6. 4-Nitro-Ethyl Cinnamate 8
Table S7. 3-Nitro-Cinnamic Acid 9
Table S8. 3-Nitro-Cinnamic Acid, Protonated 10
Table S9. 3-Nitro-Ethyl Cinnamate 11
Table S10. 4-Fluoro-Cinnamic Acid 12
Table S11. 4-Fluoro-Cinnamic Acid, Protonated 13
Table S12. 4-Fluoro-Ethyl Cinnamate 14
Table S13. 3-Fluoro-Cinnamic Acid 15
Table S14. 3-Fluoro-Cinnamic Acid, Protonated 16
Table S15. 3-Fluoro-Ethyl Cinnamate 17
Table S16. 4-Chloro-Cinnamic Acid 18
Table S17. 4-Chloro-Cinnamic Acid, Protonated 19
Table S18. 4-Chloro-Ethyl Cinnamate 20
Table S19. 4-Cyano-Cinnamic Acid 21
Table S20. 4-Cyano-Cinnamic Acid, Protonated 22
Table S21. 4-Cyano-Ethyl Cinnamate 23
Table S22. 4-Methoxy-Cinnamic Acid 24
Table S23. 4-Methoxy-Cinnamic Acid, Protonated 25
Table S24. 4-Methoxy-Ethyl Cinnamate 26
Table S25. 4-Hydroxy-Cinnamic Acid 27
Table S26. 4-Hydroxy-Cinnamic Acid, Protonated 28
Table S27. 4-Hydroxy-Ethyl Cinnamate 29
Table S28. 4-Methyl-Cinnamic Acid 30
Table S29. 4-Methyl-Cinnamic Acid, Protonated 31
Table S30. 4-Methyl-Ethyl Cinnamate 32

Ethyl 3- and 4-X-Cinnamates Characterization

Ethyl cinnamate. Yield 65%; oil; IR (KBr): ν 2980, 2932, 1711, 1636, 1609, 1262, 813; ^{1}H-NMR (DMSO-d_6): δ 7.69 (d, J = 16.0 Hz, 1H) 6,44 (d, J = 15.9 Hz, 1H) 4.26 (q, 2H) 1.34(t, 3H), 6.3–7.63 (m, 5H); ^{13}C-NMR (DMSO-d_6): δ 165.9, 143.9, 133.8, 129.9, 128.5,127.8, 117.9, 59.7, 14.1.

Ethyl 4-methoxy-cinnamate. Yield 56%; m.p. 49 °C; IR (KBr): ν 3050, 2954, 1732, 1658, 1590, 1181; ^{1}H-NMR (DMSO-d_6): δ 7.62 (d, J = 15.9 Hz, 1H), 6.39 (d, J = 16 Hz, 1H), 4.10 (q, 2H), 1.30 (t, 3H), 7.28 (d, 2H), 7.45 (d, 2H), 3.76 (s, 3H); ^{13}C-NMR (DMSO-d_6): δ 167.0, 144.5, 138.2, 135.4, 131.9, 125.0, 116.1, 60.2, 55.40, 16.3.

Ethyl 4-hydroxy-cinnamate. Yield 45%; m.p. 77 °C; IR (KBr): ν 3012, 2950, 1728, 1640, 1621, 1118, 790; ^{1}H-NMR (DMSO-d_6): δ 7.60 (d, J = 16.2 Hz, 1H), 6.35 (d, J = 15.8 Hz, 1H), 4.00 (q, 2H), 1.30 (t, 3H), 6.88 (d, J = 8.80 Hz, 2H), 7.50 (d, J = 8.98 Hz, 2H); ^{13}C-NMR (DMSO-d_6): δ 206.5, 167.9, 159.6, 144.1, 130.1, 125.2, 115.7, 56.0, 18.5.

Ethyl 4-cyano-cinnamate. Yield 75%; m.p. 66–69 °C; IR (KBr): ν 2980, 2710, 2250, 1728, 1580, 1180, 875; ^{1}H-NMR (DMSO-d_6): δ 7.70 (d, J = 15.75 Hz, 1H), 6.51 (d, J = 15.6 Hz, 1H), 4.30 (q, 2H), 1.35 (t, 3H), 7.58 (d, J = 8.1 Hz, 2H), 7.66 (d, J = 8.3 Hz 2H); ^{13}C-NMR (DMSO-d_6): δ 66.2, 142.7, 140.2, 136.7, 133.2, 128.9, 123.9, 120.0, 61.2, 18.5.

Ethyl 4-nitro-cinnamate. Yield 80%; m.p. 137–140 °C; IR (KBr): ν 3231, 3075, 1744, 1638, 1595, 1496, 1425; 994, 825. ^{1}H-NMR (DMSO-d_6): δ 7.66 (d, J = 15.80 Hz, 1H), 6.45 (d, J = 15.79 Hz, 1H), 4.45 (q, 2H), 1.31 (t, 3H), 7.66 (d, J = 8.0 Hz, 1H), 7.55 (d, J = 8.1 Hz 2H); 7.51(d, J = 7.8 Hz, 1H), 7.53 (d, J= 7.9 Hz, 1H), ^{13}C-NMR (DMSO-d_6): δ 166.8, 147.6, 145.9, 142.1, 129.7, 125.1, 116.8, 60.1, 14.4.

Ethyl 4-fluoro-cinnamate. Yield 66%; IR (KBr): ν 2930, 2870, 1732, 1600, 1650, 1271, 850; ^{1}H-NMR (DMSO-d_6): δ 7.65 (d, J = 15.8 Hz, 1H), 6.36 (d, J = 15.7 Hz, 1H), 4.26 (q, 2H), 1.33 (t, 3H), 7.05 (m, 5H); ^{13}C-NMR (DMSO-d_6): δ 166.7, 166.2, 161.2, 143.1, 130.6, 129.7, 117.9, 60.4, 14.2.

Ethyl 4-chloro-cinnamate. Yield 70%; m.p. 50 °C; IR (KBr): ν 3000, 2770, 1742, 1596, 1650, 1161, 790; ^{1}H-NMR (DMSO-d_6): δ 7.65 (d, J = 15.7 Hz, 1H), 6.40 (d, J = 15.7 Hz, 1H), 4.26 (q, 2H), 1.32 (t, 3H), 7.30 (d, 2H), 7.40 (d, 2H); ^{13}C-NMR (DMSO-d_6): δ 166.7, 143.1, 136.1, 132.9, 129.1, 118.8, 60.6, 14.3.

Ethyl 4-methyl-cinnamate. Yield 58%; oil; IR (KBr): ν 2950, 2910, 1721, 1610, 1602, 1071, 810; ^{1}H-NMR (DMSO-d_6): δ 7.64 (d, J = 16.0Hz, 1H), 6.42 (d, J = 15.9Hz, 1H), 4.25(q, 2H), 1.36(t, 3H), 7.18(d, 2H), 7.35(d, 2H), 2.1(s, 3H); ^{13}C-NMR (100MHz): δ(ppm) 166.8, 144.3, 140.3, 131.5, 129.3, 127.8, 116.9, 60.1, 21.1, 14.1.

Ethyl 3-nitro-cinnamate. Yield 72%; m.p. 72–75 °C; IR (KBr): ν 3087, 3075, 1616, 1574, 1480, 1452, 923, 807; ^{1}H-NMR (DMSO-d_6): δ 8.37 (s, 1H), 8.08 (m, 1H), 4.29(q, 2H), 1.33 (t, 3H), 7.76 (m, 1H), 7.51 (t, 1H), 7.75 (d, J = 16.0 Hz, 1H) 6.39 (d, J = 16Hz, 1H); ^{13}C-NMR (DMSO-d_6): δ 166.9, 150.2, 146.8, 138.5, 134.6, 130.2, 122.62, 116.9, 60.0, 14.4.

Ethyl 3-fluoro-cinnamate. Yield 72%; IR (KBr): ν 2860, 2650, 1712, 1628, 1602, 1311, 898; ^{1}H-NMR (DMSO-d_6): δ 7.64 (d, 1H), 6.43 (d, 1H), 4.24 (q, 2H), 1.29 (t, 3H), 7.39-7.60 (m, 4H); ^{13}C-NMR (DMSO-d_6): δ 167.0, 160.4, 143.1, 140.3, 136.6, 123.9, 116.8, 60.4, 14.2.

Cinnamic Acid

Atom	X	Y	Z
C	1.59463	−0.85338	0.00110
C	2.98443	−0.77550	0.00154
C	3.62324	0.47090	0.00086
C	2.85907	1.63991	−0.00027
C	1.46618	1.56139	−0.00071
C	0.80830	0.31658	−0.00005
H	1.11784	−1.82829	0.00163
H	3.57395	−1.68761	0.00242
H	4.70776	0.52710	0.00121
H	3.34578	2.61070	−0.00080
H	0.87437	2.47307	−0.00160
C	−0.65686	0.30047	−0.00059
H	−1.13342	1.27838	−0.00144
C	−1.46161	−0.78195	−0.00005
H	−1.08171	−1.79772	0.00087
C	−2.92969	−0.71116	−0.00046
O	−3.66013	−1.68750	−0.00049
O	−3.43141	0.55666	−0.00072
H	−4.39844	0.46747	−0.00080

Frequencies (cm^{-1}): 36.29 85.71 105.39 132.95 234.84 281.56 342.33 414.19 492.16 516.48 566.30 581.16 631.59 650.09 691.62 723.13 783.72 851.98 856.31 897.96 917.92 935.46 984.69 1004.98 1013.12 1024.45 1051.11 1106.71 1178.38 1186.56 1208.29 1237.17 1293.69 1332.91 1359.28 1366.42 1379.19 1484.87 1532.02 1622.18 1648.29 1684.62 1779.28 3179.07 3182.17 3186.80 3195.68 3203.67 3211.02 3219.15 3773.15

E = −498.263374450 hartrees

Cinnamic Acid, Protonated

Atom	X	Y	Z
C	1.63317	−0.83312	−0.01039
C	3.01560	−0.74821	−0.03189
C	3.64277	0.50893	−0.06126
C	2.88453	1.68595	−0.06918
C	1.49841	1.60863	−0.04775
C	0.84510	0.34858	−0.01794
H	1.16051	−1.80921	0.01221
H	3.61540	−1.65216	−0.02607
H	4.72684	0.56674	−0.07798
H	3.37687	2.65227	−0.09191
H	0.90321	2.51741	−0.05372
C	−0.58417	0.33694	0.00307
H	−1.06412	1.31390	−0.00569
C	−1.41159	−0.76850	0.03296
H	−1.03839	−1.78476	0.04392
C	−2.80655	−0.64494	0.05128
O	−3.37747	0.54377	0.04053
H	−4.34808	0.54852	0.05444
O	−3.51678	−1.75299	0.07941
H	−4.48196	−1.64459	0.09186

Frequencies (cm^{-1}): 7.70 73.61 95.36 104.71 199.15 237.42 322.64 338.77 399.87 439.59 472.47 535.53 565.36 623.01 647.40 663.34 706.01 779.28 843.46 865.61 875.98 958.29 988.04 998.19 1009.31 1018.91 1030.53 1043.77 1117.75 1121.23 1156.26 1197.93 1213.77 1251.67 1309.28 1330.81 1369.86 1388.86 1480.67 1498.19 1523.81 1593.88 1610.22 1629.73 1659.56 3181.20 3197.65 3204.91 3210.97 3221.58 3226.66 3242.74 3766.76 3780.58

E = −498.598846693 hartrees

Ethyl Cinnamate

Atom	X	Y	Z
C	2.27969	−1.46158	−0.00037
C	3.66555	−1.59397	−0.00047
C	4.48543	−0.45849	−0.00034
C	3.90559	0.81201	−0.00011
C	2.51653	0.94391	0.00000
C	1.67788	−0.18694	−0.00013
H	1.66179	−2.35380	−0.00047
H	4.11071	−2.58467	−0.00065
H	5.56605	−0.56652	−0.00042
H	4.53265	1.69873	0.00000
H	2.06884	1.93444	0.00018
C	0.22587	0.01815	0.00000
H	−0.09843	1.05644	0.00017
C	−0.73124	−0.93075	−0.00010
H	−0.50289	−1.99140	−0.00029
C	−2.18054	−0.65477	−0.00001
O	−3.02799	−1.53323	−0.00020
O	−2.48528	0.66628	0.00029
C	−3.89465	0.99715	0.00032
H	−4.35944	0.54874	0.88387
H	−4.35941	0.54925	−0.88351
C	−4.01133	2.50958	0.00081
H	−3.53913	2.94132	−0.88701
H	−5.06817	2.79646	0.00087
H	−3.53918	2.94077	0.88893

Frequencies (cm^{-1}): 20.91 59.46 65.92 68.74 105.17 159.92 172.86 248.93 258.62 280.70 344.35 380.98 411.96 494.48 508.85 600.50 632.18 691.47 709.71 720.49 781.69 811.38 850.75 853.30 877.82 897.25 933.85 975.41 983.05 1002.99 1012.81 1020.62 1051.11 1071.81 1106.43 1134.59 1178.16 1185.90 1206.79 1229.80 1282.12 1294.81 1297.24 1332.74 1364.68 1366.70 1400.08 1432.12 1484.27 1491.48 1503.26 1521.90 1531.94 1622.01 1648.40 1685.31 1759.01 3048.23 3064.90 3104.10 3120.09 3131.17 3177.57 3182.09 3185.04 3194.45 3202.14 3209.26 3213.69

E = −576.889296944 hartrees

4-Nitro-Cinnamic Acid

Atom	X	Y	Z
C	−0.23250	−1.02393	−0.00007
C	−1.61410	−1.16531	−0.00004
C	−2.41002	−0.01690	0.00001
C	−1.85685	1.26217	0.00003
C	−0.47062	1.38616	0.00000
C	0.36655	0.25310	−0.00005
H	0.38372	−1.91628	−0.00011
H	−2.08279	−2.14161	−0.00006
H	−2.50537	2.12945	0.00008
H	−0.02571	2.37686	0.00002
C	1.81851	0.45990	−0.00008
H	2.14333	1.49770	−0.00006
C	2.77017	−0.49355	−0.00015
H	2.54448	−1.55444	−0.00016
C	4.21698	−0.20840	−0.00021
O	4.52267	1.11686	0.00012
H	5.49205	1.17996	0.00032
O	5.07506	−1.07207	0.00005
N	−3.87573	−0.16270	0.00004
O	−4.55364	0.86610	0.00014
O	−4.33762	−1.30489	0.00007

Frequencies (cm^{-1}): 27.74 52.50 58.35 83.48 122.71 165.67 198.73 236.83 328.77 330.24 416.84 437.28 489.66 509.79 542.55 571.40 609.31 644.88 677.39 687.33 725.91 764.24 836.57 845.11 864.44 877.02 912.23 921.32 985.78 996.34 1023.45 1028.19 1124.18 1132.60 1180.26 1208.31 1236.62 1295.29 1324.77 1354.33 1365.41 1379.43 1385.32 1448.23 1530.16 1589.58 1644.68 1646.82 1688.99 1786.49 3186.29 3197.89 3210.13 3220.95 3242.65 3243.34 3770.59

E = −702.770541159 hartrees

4-Nitro-Cinnamic Acid, Protonated

Atom	X	Y	Z
C	−0.27757	−1.01919	−0.00003
C	−1.65670	−1.15618	−0.00004
C	−2.44306	−0.00016	−0.00002
C	−1.90291	1.28416	0.00003
C	−0.51937	1.41270	0.00004
C	0.31622	0.26843	0.00001
H	0.33637	−1.91292	−0.00005
H	−2.13417	−2.12854	−0.00008
H	−2.55926	2.14591	0.00005
H	−0.07481	2.40342	0.00008
C	1.73866	0.47805	0.00003
H	2.06143	1.51759	0.00006
C	2.71879	−0.48689	0.00000
H	2.50596	−1.54870	−0.00003
C	4.08645	−0.15211	0.00003
O	4.46503	1.10616	0.00008
H	5.42322	1.26720	0.00006
O	4.94778	−1.14124	−0.00003
H	5.88948	−0.89942	0.00003
N	−3.92474	−0.14961	−0.00004
O	−4.58733	0.88245	−0.00001
O	−4.36774	−1.29363	−0.00006

Frequencies (cm^{-1}): 33.05 46.59 47.46 83.08 128.98 168.33 200.46 236.17 266.79 325.44 353.35 410.76 433.53 472.44 486.34 513.34 549.88 597.73 638.83 662.62 688.24 710.55 749.18 838.94 840.60 868.49 880.35 892.77 992.09 993.92 1003.88 1023.30 1024.44 1115.48 1119.91 1136.05 1154.65 1209.30 1250.37 1305.32 1325.13 1352.15 1380.17 1389.25 1442.52 1510.70 1522.97 1588.13 1602.95 1635.25 1652.83 1656.57 3183.26 3205.29 3217.37 3241.19 3244.99 3245.73 3756.26 3769.99

E = −703.090479796 hartrees

4-Nitro-Ethyl Cinnamate

Atom	X	Y	Z
C	−1.11607	−1.30081	0.00000
C	−2.50129	−1.39985	0.00004
C	−3.26235	−0.22790	0.00001
C	−2.66952	1.03339	−0.00008
C	−1.28015	1.11466	−0.00013
C	−0.47711	−0.04307	−0.00009
H	−0.52779	−2.21189	0.00004
H	−2.99940	−2.36146	0.00011
H	−3.29099	1.92026	−0.00012
H	−0.80480	2.09118	−0.00020
C	0.98111	0.11884	−0.00013
H	1.33919	1.14558	−0.00021
C	1.90108	−0.86417	−0.00009
H	1.63632	−1.91634	0.00000
C	3.36415	−0.64028	−0.00016
O	3.71356	0.66527	−0.00007
O	4.17144	−1.55407	0.00017
N	−4.73082	−0.32877	0.00006
O	−5.37786	0.72012	−0.00003
O	−5.22802	−1.45639	0.00007
C	5.13675	0.94789	0.00018
H	5.58272	0.48244	−0.88387
H	5.58241	0.48254	0.88444
C	5.30559	2.45481	0.00011
H	4.85017	2.90280	0.88851
H	6.37196	2.70299	−0.00016
H	4.84973	2.90278	−0.88807

Frequencies (cm^{-1}): 18.72 39.94 56.19 57.05 61.60 91.52 130.79 155.81 179.10 225.62 240.27 261.06 330.88 331.01 379.19 417.28 434.51 492.65 507.40 540.48 643.96 656.97 675.31 718.02 724.81 762.79 810.95 836.45 844.44 863.62 869.56 882.93 912.33 976.72 985.06 995.69 1019.66 1028.62 1066.80 1125.76 1133.50 1134.78 1177.82 1208.98 1230.58 1280.52 1295.78 1301.02 1326.09 1350.92 1376.52 1379.40 1400.67 1432.19 1448.49 1491.60 1502.88 1521.01 1530.62 1587.94 1644.29 1646.17 1688.35 1764.47 3049.92 3068.75 3108.51 3122.60 3133.82 3186.00 3196.76 3207.65 3217.52 3242.77 3243.10

E = −781.397275781 hartrees

3-Nitro-Cinnamic Acid

Atom	X	Y	Z
C	−0.72767	−0.27772	−0.00002
C	−2.09957	−0.05989	−0.00002
C	−2.66072	1.21906	0.00001
C	−1.79774	2.31314	0.00004
C	−0.41553	2.11833	0.00003
C	0.14368	0.82573	0.00000
H	−0.36128	−1.29628	−0.00004
H	−2.20368	3.31946	0.00007
H	0.24691	2.97946	0.00005
C	1.60472	0.68838	0.00000
H	2.15981	1.62338	0.00005
C	2.31102	−0.45823	−0.00007
H	1.84732	−1.43890	−0.00013
C	3.78405	−0.51031	−0.00008
O	4.38438	0.71154	0.00009
H	5.34223	0.55007	0.00016
O	4.42533	−1.54506	−0.00003
H	−3.73713	1.33543	0.00002
N	−3.00135	−1.23120	−0.00004
O	−4.21371	−1.01700	0.00002
O	−2.48826	−2.35012	0.00003

Frequencies (cm^{-1}): 25.73 45.91 77.83 85.40 122.44 173.27 187.09 262.31 279.21 358.74 393.56 429.56 496.16 517.62 552.56 572.25 583.79 646.67 668.40 696.90 726.55 755.82 819.20 832.87 900.08 918.09 933.38 954.60 956.44 1004.34 1014.81 1021.35 1107.02 1118.40 1179.64 1194.84 1234.98 1292.98 1318.85 1352.90 1365.83 1380.80 1386.44 1474.98 1515.74 1603.00 1623.71 1664.41 1690.52 1787.72 3185.41 3192.11 3211.90 3218.60 3246.78 3247.07 3771.08

E = −702.769955161 hartrees

3-Nitro-Cinnamic Acid, Protonated

Atom	X	Y	Z
C	−0.76473	−0.25637	0.00000
C	−2.12851	−0.03430	0.00000
C	−2.68146	1.25078	0.00004
C	−1.82414	2.35475	0.00007
C	−0.44661	2.16365	0.00006
C	0.10690	0.85797	0.00003
H	−0.40709	−1.27845	−0.00002
H	−2.23682	3.35759	0.00009
H	0.21699	3.02355	0.00009
C	1.53698	0.72120	0.00003
H	2.09975	1.65276	0.00006
C	2.25496	−0.45246	0.00000
H	1.79084	−1.43113	−0.00003
C	3.66219	−0.45861	−0.00001
O	4.33444	0.67132	−0.00009
H	5.30293	0.59429	0.00008
O	4.25946	−1.62648	0.00001
H	5.23163	−1.61881	−0.00009
H	−3.75942	1.36628	0.00004
N	−3.03700	−1.20998	−0.00002
O	−4.24132	−0.98382	0.00000
O	−2.51174	−2.32050	−0.00009

Frequencies (cm^{-1}): 39.70 46.93 77.23 82.43 151.66 172.42 187.68 243.38 261.73 336.87 354.58 389.39 426.53 478.10 505.01 507.67 557.92 572.42 643.76 656.76 695.27 713.10 739.20 822.19 834.97 888.07 935.05 959.77 968.83 993.02 1011.56 1020.09 1026.67 1110.83 1116.39 1120.98 1156.02 1200.99 1248.40 1302.22 1315.91 1350.00 1385.16 1390.30 1476.97 1500.42 1515.44 1588.38 1608.43 1621.98 1647.86 1672.97 3184.66 3201.91 3225.47 3237.15 3240.63 3247.53 3757.39 3771.28

E = −703.092895283 hartrees

3-Nitro-Ethyl Cinnamate

Atom	X	Y	Z
C	−1.60191	−0.40633	0.00024
C	−2.96242	−0.12523	0.00031
C	−3.46402	1.17821	0.00008
C	−2.55064	2.23051	−0.00023
C	−1.17880	1.97170	−0.00030
C	−0.67938	0.65491	−0.00007
H	−1.28367	−1.44098	0.00042
H	−2.90935	3.25470	−0.00041
H	−0.47719	2.80123	−0.00054
C	0.77458	0.44962	−0.00016
H	1.37417	1.35668	−0.00043
C	1.42548	−0.72827	0.00006
H	0.91164	−1.68397	0.00035
C	2.89851	−0.86463	−0.00005
O	3.55303	0.31973	−0.00022
O	3.46413	−1.94457	0.00033
H	−4.53380	1.34452	0.00015
N	−3.91744	−1.25333	0.00063
O	−5.11892	−0.98338	0.00069
O	−3.45732	−2.39524	0.00082
C	5.00146	0.25036	−0.00024
H	5.32228	−0.30962	−0.88375
H	5.32233	−0.30787	0.88437
C	5.52939	1.67198	−0.00160
H	5.19527	2.21773	0.88603
H	6.62414	1.65572	−0.00154
H	5.19534	2.21597	−0.89032

Frequencies (cm^{-1}): 13.92 44.58 57.01 61.46 70.24 102.09 113.30 162.47 179.54 234.29 256.08 256.76 281.40 355.11 379.65 397.37 430.24 489.80 523.98 550.40 602.56 667.46 684.55 718.27 724.53 754.68 811.33 819.49 834.17 871.44 900.14 932.37 954.35 955.96 975.91 1003.98 1014.30 1019.87 1067.77 1106.25 1118.11 1133.89 1177.33 1194.19 1228.08 1277.84 1294.37 1299.40 1317.82 1349.88 1373.77 1384.94 1400.08 1431.94 1474.06 1490.49 1503.59 1515.81 1520.71 1602.36 1623.03 1664.00 1690.91 1765.34 3049.50 3068.15 3107.68 3122.29 3133.08 3185.78 3191.84 3210.27 3211.46 3244.30 3246.27

E = −781.396451200 hartrees

4-Fluoro-Cinnamic Acid

Atom	X	Y	Z
C	1.13392	−0.90385	0.00096
C	2.52331	−0.83589	0.00135
C	3.12938	0.41814	0.00067
C	2.39730	1.59842	−0.00040
C	1.00588	1.51026	−0.00078
C	0.34619	0.26587	−0.00010
H	0.65993	−1.87974	0.00151
H	3.13664	−1.73037	0.00217
H	2.90938	2.55434	−0.00091
H	0.41741	2.42355	−0.00161
C	−1.11781	0.24958	−0.00057
H	−1.59401	1.22762	−0.00135
C	−1.92324	−0.83212	−0.00010
H	−1.54449	−1.84849	0.00073
C	−3.39126	−0.75968	−0.00052
O	−3.89068	0.50909	−0.00109
H	−4.85797	0.42236	−0.00143
O	−4.12200	−1.73543	−0.00037
F	4.48362	0.48632	0.00106

Frequencies (cm^{-1}): 30.11 65.89 94.98 123.46 205.65 220.59 312.91 373.52 413.88 418.76 509.36 512.62 539.92 565.68 626.46 648.85 689.38 747.18 795.81 825.41 848.62 872.80 897.06 918.86 955.65 973.14 1021.46 1028.33 1124.73 1176.47 1185.36 1232.86 1261.05 1294.21 1323.57 1344.10 1360.15 1379.44 1450.62 1546.17 1629.70 1649.16 1686.42 1780.68 3181.32 3191.99 3203.36 3216.60 3222.11 3223.33 3772.91

E = −597.503464674 hartrees

4-Fluoro-Cinnamic Acid, Protonated

Atom	X	Y	Z
C	1.17864	−0.88666	−0.00010
C	2.55816	−0.81273	−0.02096
C	3.15802	0.45354	−0.05278
C	2.42652	1.64305	−0.06463
C	1.04471	1.55569	−0.04378
C	0.38751	0.29552	−0.01198
H	0.70795	−1.86328	0.02541
H	3.18215	−1.69931	−0.01267
H	2.94472	2.59491	−0.08917
H	0.45250	2.46598	−0.05233
C	−1.03883	0.28315	0.00698
H	−1.51908	1.25986	0.00007
C	−1.86822	−0.82230	0.03376
H	−1.49691	−1.83937	0.03637
C	−3.26206	−0.69716	0.05867
O	−3.83110	0.49275	0.08902
H	−4.80071	0.49971	0.04257
O	−3.97495	−1.80431	0.05513
H	−4.93803	−1.69470	0.11769
F	4.48989	0.52178	−0.07248

Frequencies (cm^{-1}): 29.66 57.28 94.09 103.97 164.37 223.19 267.92 312.11 367.57 409.13 420.70 440.33 514.60 517.41 539.91 618.38 641.43 682.85 721.68 809.21 821.65 858.18 871.53 877.25 971.99 984.77 988.42 1015.82 1019.18 1122.83 1136.14 1154.98 1190.39 1247.42 1300.42 1311.00 1328.23 1343.34 1385.63 1466.44 1495.54 1538.86 1593.55 1607.73 1627.52 1667.88 3182.36 3204.31 3215.04 3233.27 3233.92 3241.75 3767.66 3780.79

E = −597.836966323 hartrees

4-Fluoro-Ethyl Cinnamate

Atom	X	Y	Z
C	1.85185	−1.37818	−0.00019
C	3.23846	−1.49277	−0.00034
C	4.00352	−0.32914	−0.00025
C	3.43209	0.93635	−0.00002
C	2.04063	1.03093	0.00013
C	1.22330	−0.11584	0.00005
H	1.25417	−2.28362	−0.00025
H	3.72932	−2.45992	−0.00052
H	4.06492	1.81702	0.00004
H	1.57652	2.01330	0.00031
C	−0.23171	0.05923	0.00022
H	−0.57703	1.09066	0.00040
C	−1.16976	−0.90838	0.00015
H	−0.92168	−1.96463	−0.00005
C	−2.62429	−0.66042	0.00033
O	−2.95412	0.65396	0.00028
O	−3.45355	−1.55598	−0.00004
F	5.35617	−0.43844	−0.00040
C	−4.37027	0.95762	0.00010
H	−4.82568	0.49999	0.88374
H	−4.82545	0.49997	−0.88365
C	−4.51626	2.46725	0.00006
H	−4.05289	2.90815	−0.88791
H	−5.57851	2.73327	−0.00008
H	−4.05312	2.90817	0.88814

Frequencies (cm^{-1}): 21.36 50.90 60.61 62.73 96.27 147.23 170.94 203.31 240.11 261.82 318.88 373.78 373.83 416.94 421.97 502.18 515.47 544.23 648.14 690.18 698.80 745.78 797.97 810.84 824.46 847.64 865.78 881.43 899.97 954.93 972.05 976.06 1017.30 1028.52 1071.08 1124.48 1134.47 1178.15 1182.75 1225.30 1255.98 1284.14 1295.02 1297.39 1324.30 1342.10 1365.94 1400.26 1432.06 1449.93 1491.52 1503.17 1521.89 1546.06 1630.32 1649.30 1686.30 1759.43 3048.44 3065.66 3104.80 3120.34 3131.42 3181.50 3190.77 3201.83 3212.25 3221.75 3222.39

E = −676.129492922 hartrees

3-Fluoro-Cinnamic Acid

Atom	X	Y	Z
C	1.23892	−0.67993	0.00081
C	2.61878	−0.57233	0.00113
C	3.28536	0.65047	0.00051
C	2.51522	1.81463	−0.00049
C	1.12148	1.73987	−0.00084
C	0.46232	0.49589	−0.00020
H	0.78931	−1.66622	0.00133
H	3.00607	2.78281	−0.00099
H	0.53217	2.65209	−0.00162
C	−1.00357	0.48004	−0.00061
H	−1.47844	1.45831	−0.00139
C	−1.80509	−0.60351	−0.00013
H	−1.42333	−1.61871	0.00066
C	−3.27496	−0.53525	−0.00059
O	−3.77658	0.73145	−0.00164
H	−4.74388	0.64387	−0.00185
O	−4.00145	−1.51355	−0.00006
F	3.35802	−1.71224	0.00211
H	4.36946	0.67553	0.00081

Frequencies (cm^{-1}): 27.81 81.92 94.48 124.02 211.17 238.07 278.69 327.85 423.43 450.24 523.04 526.29 561.14 583.00 587.75 646.42 674.80 741.19 775.76 797.97 883.26 893.52 918.03 918.34 984.80 985.01 1016.20 1018.90 1102.59 1173.52 1178.71 1190.62 1258.26 1299.44 1314.67 1350.96 1364.44 1377.03 1483.44 1520.75 1627.72 1655.02 1686.30 1782.61 3184.76 3191.66 3206.54 3217.22 3224.78 3226.92 3772.07

E = −597.503012709 hartrees

3-Fluoro-Cinnamic Acid, Protonated

Atom	X	Y	Z
C	1.27777	−0.66662	−0.00780
C	2.65306	−0.55440	−0.02983
C	3.30658	0.68310	−0.05940
C	2.54323	1.85315	−0.06708
C	1.15407	1.77792	−0.04546
C	0.50092	0.51981	−0.01550
H	0.83311	−1.65461	0.01469
H	3.03789	2.81802	−0.08994
H	0.56136	2.68746	−0.05146
C	−0.93168	0.51004	0.00569
H	−1.40839	1.48816	−0.00321
C	−1.75559	−0.59473	0.03533
H	−1.38133	−1.61081	0.04611
C	−3.15373	−0.47327	0.05368
O	−3.72399	0.71363	0.04256
H	−4.69493	0.72057	0.05744
O	−3.85868	−1.58233	0.08178
H	−4.82494	−1.48007	0.09402
F	3.39774	−1.67325	−0.02271
H	4.39116	0.71010	−0.07579

Frequencies (cm^{-1}): 31.89 74.04 94.72 121.47 204.20 214.23 241.52 326.30 329.03 426.43 439.25 459.90 519.66 545.10 566.26 568.45 644.16 652.66 714.03 782.68 803.80 870.59 885.28 941.14 987.60 993.76 1005.10 1014.46 1016.12 1112.58 1122.02 1156.98 1190.97 1209.16 1265.50 1313.53 1318.04 1349.95 1391.31 1482.72 1497.97 1515.41 1591.78 1619.30 1635.15 1658.52 3186.10 3205.12 3220.44 3230.64 3231.20 3243.19 3763.10 3776.78

E = −597.833178389 hartrees

3-Fluoro-Ethyl Cinnamate

Atom	X	Y	Z
C	2.06888	−1.01192	0.00003
C	3.45174	−0.95226	0.00005
C	4.16108	0.24608	0.00004
C	3.43133	1.43601	0.00000
C	2.03574	1.40943	−0.00001
C	1.33287	0.18960	0.00000
H	1.58498	−1.98179	0.00005
H	3.95520	2.38682	−0.00001
H	1.47872	2.34177	−0.00004
C	−0.13362	0.22481	−0.00002
H	−0.57467	1.21869	−0.00004
C	−0.97224	−0.82917	−0.00001
H	−0.62265	−1.85645	0.00001
C	−2.44538	−0.72154	−0.00003
O	−2.89764	0.55512	−0.00004
O	−3.18433	−1.69229	0.00001
F	4.15107	−2.11785	0.00008
H	5.24538	0.23311	0.00005
C	−4.33651	0.72518	−0.00003
H	−4.74684	0.22744	−0.88400
H	−4.74683	0.22747	0.88396
C	−4.62215	2.21492	−0.00006
H	−5.70449	2.38112	−0.00005
H	−4.20149	2.69653	0.88800
H	−4.20150	2.69650	−0.88813

Frequencies (cm^{-1}): 15.68 59.43 63.68 64.04 102.19 136.73 171.56 238.49 242.31 259.74 281.19 329.90 371.48 427.88 453.66 517.93 524.20 581.86 600.99 675.38 704.18 738.61 780.87 797.30 811.90 871.51 883.99 895.88 918.34 975.63 984.15 984.96 1016.08 1018.72 1069.10 1102.38 1134.04 1170.83 1177.51 1189.83 1243.47 1293.66 1293.67 1300.81 1315.58 1350.65 1367.13 1398.93 1430.90 1482.71 1491.03 1503.46 1519.58 1521.51 1627.10 1654.83 1686.95 1761.38 3048.36 3066.07 3105.63 3120.65 3131.88 3184.04 3190.54 3205.67 3211.74 3224.46 3226.16

E = −676.129166341 hartrees

4-Chloro-Cinnamic Acid

Atom	X	Y	Z
C	0.67536	−0.94680	0.00081
C	2.06437	−0.87890	0.00118
C	2.69028	0.37193	0.00054
C	1.94158	1.54848	−0.00045
C	0.55029	1.46366	−0.00080
C	−0.11217	0.22194	−0.00018
H	0.20270	−1.92341	0.00131
H	2.43868	2.51212	−0.00093
H	−0.03482	2.37923	−0.00158
C	−1.57638	0.20742	−0.00061
H	−2.05203	1.18560	−0.00137
C	−2.38104	−0.87481	−0.00014
H	−2.00196	−1.89098	0.00064
C	−3.84977	−0.80336	−0.00059
O	−4.34929	0.46465	−0.00166
H	−5.31668	0.37850	−0.00186
O	−4.57922	−1.77991	−0.00006
H	2.66136	−1.78418	0.00195
Cl	4.44210	0.45928	0.00100

Frequencies (cm^{-1}): 33.01 56.52 87.42 126.06 178.54 207.03 265.41 348.89 349.28 416.66 451.32 502.32 524.00 567.94 611.77 646.12 695.33 701.32 748.03 832.58 838.65 858.61 902.49 919.27 963.43 977.75 1022.18 1025.61 1106.56 1134.18 1179.03 1207.35 1238.30 1293.92 1325.60 1335.68 1359.18 1377.01 1442.51 1527.91 1606.27 1639.27 1685.45 1780.88 3182.56 3190.35 3202.46 3217.20 3221.05 3222.41 3772.52

E = −957.857121709 hartrees

4-Chloro-Cinnamic Acid, Protonated

Atom	X	Y	Z
C	0.72424	−0.93124	0.00790
C	2.10370	−0.85699	−0.01293
C	2.72833	0.40481	−0.04513
C	1.97566	1.58895	−0.05647
C	0.59365	1.50570	−0.03572
C	−0.06723	0.24910	−0.00390
H	0.25543	−1.90894	0.03349
H	2.47660	2.54984	−0.08098
H	0.00535	2.41874	−0.04436
C	−1.49369	0.23866	0.01532
H	−1.97316	1.21572	0.00877
C	−2.32276	−0.86722	0.04111
H	−1.95027	−1.88387	0.04463
C	−3.71656	−0.74357	0.06441
O	−4.28696	0.44611	0.08560
H	−5.25709	0.45126	0.05359
O	−4.42863	−1.85163	0.06750
H	−5.39231	−1.74182	0.11804
H	2.70894	−1.75629	−0.00413
Cl	4.45289	0.49237	−0.07067

Frequencies (cm^{-1}): 22.51 48.02 87.49 115.39 146.79 209.84 247.71 268.62 350.32 356.18 417.75 436.68 455.68 502.83 538.90 601.32 638.68 685.49 713.15 722.49 828.57 843.72 867.41 880.65 978.39 988.03 989.72 1017.80 1020.56 1110.15 1123.08 1144.86 1161.53 1215.17 1253.60 1310.28 1328.29 1337.45 1376.05 1457.94 1493.73 1520.62 1571.17 1599.23 1624.83 1661.59 3182.83 3201.19 3212.49 3230.94 3232.46 3241.47 3769.11 3781.85

E = −958.190373259 hartrees

4-Chloro-Ethyl Cinnamate

Atom	X	Y	Z
C	−1.41733	−1.31716	−0.00002
C	−2.80508	−1.41096	−0.00005
C	−3.57193	−0.24147	−0.00010
C	−2.96459	1.01363	−0.00011
C	−1.57252	1.09052	−0.00008
C	−0.76992	−0.06544	−0.00003
H	−0.83482	−2.23261	0.00002
H	−3.56991	1.91339	−0.00015
H	−1.09734	2.06777	−0.00009
C	0.68738	0.08973	0.00001
H	1.04757	1.11600	−0.00002
C	1.60995	−0.89244	0.00007
H	1.34506	−1.94459	0.00010
C	3.06891	−0.66777	0.00010
O	3.41985	0.64074	0.00008
O	3.88288	−1.57700	0.00016
H	−3.29293	−2.37952	−0.00004
Cl	−5.32305	−0.35812	−0.00014
C	4.84114	0.92149	0.00012
H	5.28869	0.45686	0.88404
H	5.28876	0.45681	−0.88373
C	5.01164	2.42875	0.00008
H	6.07812	2.67714	0.00011
H	4.55549	2.87677	−0.88804
H	4.55544	2.87681	0.88816

Frequencies (cm^{-1}): 24.38 43.43 58.40 60.70 93.54 136.86 166.56 181.16 231.38 261.36 270.46 346.16 348.93 382.61 417.06 449.08 504.80 518.52 645.43 666.67 693.50 724.57 746.42 810.60 832.21 838.11 854.99 877.79 902.51 962.47 975.71 977.02 1018.14 1025.69 1070.11 1106.55 1133.76 1135.48 1178.09 1207.06 1230.09 1282.29 1295.00 1297.01 1326.62 1334.59 1361.10 1399.88 1431.39 1442.37 1491.38 1503.01 1521.60 1528.41 1606.87 1639.48 1685.73 1759.99 3048.54 3066.26 3105.58 3120.75 3131.85 3181.88 3188.69 3200.45 3212.62 3219.23 3221.41

E = −1036.48327719 hartrees

4-Cyano-Cinnamic Acid

Atom	X	Y	Z
C	0.81851	−0.93355	0.00082
C	2.20512	−0.87234	0.00114
C	2.85627	0.37652	0.00059
C	2.09602	1.55758	−0.00030
C	0.70708	1.48265	−0.00062
C	0.04037	0.24227	−0.00007
H	0.33766	−1.90574	0.00125
H	2.59582	2.52027	−0.00073
H	0.12440	2.39933	−0.00131
C	−1.42550	0.23685	−0.00044
H	−1.89651	1.21704	−0.00113
C	−2.23140	−0.84318	−0.00003
H	−1.85542	−1.86050	0.00062
C	−3.70280	−0.76868	−0.00059
O	−4.19686	0.49941	−0.00240
H	−5.16504	0.42033	−0.00268
O	−4.42990	−1.74565	0.00056
H	2.79395	−1.78337	0.00183
C	4.28904	0.44072	0.00092
N	5.45187	0.49277	0.00120

Frequencies (cm^{-1}): 31.51 53.77 83.05 122.05 152.69 160.31 223.35 295.96 324.74 410.11 462.23 479.31 523.09 556.62 559.07 575.97 619.62 660.17 703.54 750.98 757.54 846.90 850.85 864.03 909.09 920.77 977.43 987.59 1023.08 1032.72 1140.51 1180.27 1203.33 1229.35 1240.90 1294.28 1332.14 1336.55 1360.88 1379.56 1448.56 1543.25 1594.04 1653.64 1687.81 1784.98 2334.01 3185.53 3193.95 3204.57 3217.12 3217.93 3222.72 3770.76

E = −590.506145350 hartrees

4-Cyano-Cinnamic Acid, Protonated

Atom	X	Y	Z
C	0.86506	−0.91702	0.00460
C	2.24604	−0.84831	−0.01360
C	2.88654	0.41063	−0.04232
C	2.12966	1.59754	−0.05256
C	0.74562	1.52199	−0.03421
C	0.08479	0.26799	−0.00536
H	0.38991	−1.89151	0.02661
H	2.63228	2.55791	−0.07462
H	0.16002	2.43649	−0.04207
C	−1.34914	0.26486	0.01233
H	−1.82260	1.24481	0.00212
C	−2.17796	−0.83526	0.04046
H	−1.81040	−1.85374	0.05249
C	−3.57729	−0.70651	0.05565
O	−4.14001	0.48283	0.04325
H	−5.11115	0.49722	0.05500
O	−4.28584	−1.81213	0.08239
H	−5.25209	−1.70781	0.09262
H	2.84405	−1.75271	−0.00603
C	4.31715	0.47693	−0.06111
N	5.47913	0.52952	−0.07638

Frequencies (cm^{-1}): 38.35 46.97 82.66 117.48 149.82 158.24 225.56 238.32 320.84 330.91 400.59 443.10 463.09 481.47 529.56 557.13 558.54 610.64 653.65 695.96 741.87 755.77 841.45 855.71 871.58 886.99 989.22 990.79 997.35 1022.18 1026.29 1120.63 1147.82 1155.06 1207.87 1233.78 1255.76 1308.12 1330.80 1341.78 1375.08 1453.49 1505.27 1532.59 1570.76 1600.29 1633.01 1662.48 2340.85 3183.76 3203.41 3213.99 3229.60 3230.47 3241.62 3760.17 3773.74

E = −590.829657607 hartrees

4-Cyano-Ethyl Cinnamate

Atom	X	Y	Z
C	−1.57109	−1.33420	−0.00032
C	−2.95564	−1.43247	−0.00045
C	−3.74628	−0.26685	−0.00040
C	−3.12581	0.99326	−0.00021
C	−1.73739	1.07794	−0.00008
C	−0.93200	−0.07728	−0.00013
H	−0.98214	−2.24508	−0.00036
H	−3.73244	1.89253	−0.00017
H	−1.26347	2.05538	0.00006
C	0.52560	0.08545	0.00002
H	0.88220	1.11273	0.00013
C	1.44832	−0.89547	0.00004
H	1.18544	−1.94810	−0.00007
C	2.90956	−0.66844	0.00015
O	3.25641	0.63897	0.00044
O	3.72085	−1.57893	0.00004
H	−3.43590	−2.40519	−0.00060
C	−5.17685	−0.36700	−0.00054
N	−6.33811	−0.44821	−0.00065
C	4.67806	0.92483	0.00057
H	5.12584	0.46049	0.88442
H	5.12587	0.46097	−0.88351
C	4.84359	2.43219	0.00095
H	4.38669	2.87894	0.88925
H	5.90935	2.68331	0.00108
H	4.38681	2.87937	−0.88720

Frequencies (cm^{-1}): 20.96 41.14 57.54 59.31 89.90 123.21 144.86 172.86 182.60 235.66 260.15 294.16 330.01 378.95 409.45 464.26 473.75 522.55 558.75 561.51 659.30 689.59 700.18 754.87 758.23 810.92 846.28 849.83 860.10 878.84 908.62 976.30 976.55 986.74 1019.33 1032.93 1068.31 1134.29 1141.54 1177.94 1203.13 1227.32 1236.45 1281.45 1295.75 1299.80 1331.78 1336.33 1364.51 1400.65 1432.45 1448.75 1491.60 1502.97 1521.34 1543.64 1594.05 1653.47 1687.59 1763.63 2333.06 3049.45 3067.62 3107.20 3121.85 3133.01 3185.21 3192.53 3202.65 3213.02 3217.22 3220.78

E = −669.132662051 hartrees

4-Methoxy-Cinnamic Acid

Atom	X	Y	Z
C	0.60981	−1.07651	0.17242
C	1.98751	−1.10806	0.04523
C	2.69814	0.06417	−0.27644
C	2.00257	1.26488	−0.46753
C	0.61283	1.27856	−0.33529
C	−0.11692	0.12172	−0.01511
H	0.08756	−1.99474	0.42112
H	2.52237	2.18220	−0.71579
H	0.08137	2.21481	−0.48532
C	−1.56867	0.21277	0.10645
H	−1.98884	1.20152	−0.06563
C	−2.43238	−0.78219	0.40526
H	−2.11261	−1.80117	0.59387
C	−3.88418	−0.60050	0.50765
O	−4.31107	0.67532	0.27383
H	−5.27700	0.65898	0.37151
O	−4.67071	−1.49370	0.77733
H	2.54441	−2.02808	0.18928
O	4.04794	−0.07447	−0.37778
C	4.83379	1.06918	−0.70084
H	4.73092	1.85046	0.06195
H	5.86643	0.72039	−0.72618
H	4.56099	1.47442	−1.68275

Frequencies (cm^{-1}): 34.15 55.72 85.85 102.14 132.71 194.69 213.78 228.10 248.02 349.82 375.79 427.10 448.44 521.25 527.85 556.69 562.31 627.86 651.62 698.56 748.17 780.23 818.80 845.07 867.66 892.79 917.25 950.11 974.53 1020.31 1021.78 1061.54 1138.82 1169.97 1176.13 1202.40 1205.59 1241.69 1289.14 1303.39 1329.77 1349.18 1357.80 1384.89 1462.34 1478.91 1497.54 1507.25 1550.35 1608.93 1653.77 1679.90 1775.01 3022.78 3089.01 3158.36 3177.76 3184.75 3197.49 3214.28 3218.98 3226.65 3774.33

E = −612.793992714 hartrees

4-Methoxy-Cinnamic Acid, Protonated

Atom	X	Y	Z
C	0.66252	−1.05828	0.16299
C	2.02828	−1.07410	0.03646
C	2.72968	0.11884	−0.28766
C	2.02196	1.32731	−0.48243
C	0.64700	1.32899	−0.35334
C	−0.07858	0.14807	−0.03035
H	0.14928	−1.98044	0.41312
H	2.54237	2.24392	−0.72919
H	0.10303	2.25754	−0.50259
C	−1.48621	0.23172	0.08511
H	−1.92027	1.21635	−0.07783
C	−2.37130	−0.79947	0.38254
H	−2.04865	−1.81986	0.54608
C	−3.74025	−0.58277	0.48621
O	−4.24747	0.64036	0.40513
H	−5.20456	0.67387	0.24783
O	−4.52834	−1.62843	0.67938
H	−5.43961	−1.41660	0.93859
H	2.60294	−1.98244	0.17991
O	4.04982	−0.00793	−0.38464
C	4.87611	1.12764	−0.70883
H	4.77982	1.90228	0.05738
H	5.89417	0.74316	−0.72180
H	4.61409	1.52251	−1.69464

Frequencies (cm^{-1}): 44.40 60.05 86.02 116.89 157.22 196.00 213.78 225.08 232.28 283.82 345.53 374.03 413.69 435.14 452.91 520.56 538.04 551.32 616.28 639.91 681.13 724.85 787.73 814.48 855.08 868.30 871.90 970.77 982.76 989.42 1007.32 1009.78 1026.77 1137.91 1146.74 1158.77 1164.49 1204.26 1207.03 1248.67 1308.16 1330.65 1341.54 1353.27 1388.46 1469.77 1481.30 1495.80 1502.70 1503.68 1544.07 1569.25 1610.94 1627.07 1680.63 3054.09 3136.48 3181.39 3191.35 3198.18 3211.37 3228.99 3240.55 3242.48 3772.27 3782.16

E = −613.140424751 hartrees

4-Methoxy-Ethyl Cinnamate

Atom	X	Y	Z
C	−1.31792	1.48950	−0.00003
C	−2.69176	1.65964	−0.00004
C	−3.54607	0.54096	0.00003
C	−2.99813	−0.74771	0.00009
C	−1.60995	−0.90071	0.00010
C	−0.73796	0.20023	0.00003
H	−0.68352	2.37010	−0.00009
H	−3.63084	−1.62702	0.00014
H	−1.19354	−1.90483	0.00015
C	0.70386	−0.03810	0.00005
H	1.00272	−1.08421	0.00006
C	1.68743	0.88615	0.00006
H	1.48663	1.95234	0.00007
C	3.12619	0.57239	0.00010
O	3.39756	−0.75805	−0.00002
O	3.99978	1.42594	0.00005
H	−3.13528	2.64997	−0.00009
O	−4.87982	0.81677	0.00002
C	−5.80327	−0.26755	0.00004
H	−5.68687	−0.88890	0.89637
H	−6.79404	0.18761	0.00000
H	−5.68682	−0.88897	−0.89623
C	4.79744	−1.12325	−0.00010
H	5.27400	−0.68732	−0.88370
H	5.27404	−0.68753	0.88358
C	4.87679	−2.63841	−0.00028
H	4.39367	−3.05771	−0.88826
H	5.92613	−2.95178	−0.00033
H	4.39370	−3.05791	0.88762

Frequencies (cm^{-1}): 26.92 43.15 57.29 63.03 88.82 111.54 139.64 176.68 194.42 223.36 247.31 253.20 261.15 350.21 375.01 381.42 426.50 444.36 523.43 523.85 564.64 649.08 700.84 702.86 750.12 782.13 809.94 817.86 843.95 861.43 879.00 894.28 949.10 973.60 974.78 1016.36 1022.99 1063.06 1072.89 1133.74 1139.56 1172.52 1178.25 1200.79 1207.22 1231.06 1282.02 1288.28 1293.38 1305.90 1330.64 1347.33 1371.40 1399.25 1430.38 1461.80 1481.24 1490.63 1499.29 1502.93 1509.22 1522.06 1550.69 1609.99 1654.61 1680.73 1754.79 3022.37 3047.28 3063.82 3088.13 3102.62 3119.12 3129.97 3157.07 3176.19 3182.64 3197.47 3211.80 3216.33 3224.82

E = −691.419662525 hartrees

4-Hydroxy-Cinnamic Acid

Atom	X	Y	Z
C	1.11199	−0.92508	−0.00006
C	2.49786	−0.88137	−0.03154
C	3.15670	0.35667	−0.07857
C	2.41490	1.54260	−0.09375
C	1.02339	1.48366	−0.06163
C	0.33784	0.25583	−0.01414
H	0.62276	−1.89301	0.03618
H	2.92053	2.50485	−0.13046
H	0.45462	2.40957	−0.07368
C	−1.12219	0.26452	0.01771
H	−1.58178	1.25056	0.00175
C	−1.94952	−0.80199	0.06407
H	−1.58968	−1.82492	0.08321
C	−3.41288	−0.70047	0.09356
O	−3.88988	0.57845	0.07026
H	−4.85797	0.50654	0.09326
O	−4.16620	−1.65928	0.13508
H	3.08938	−1.79075	−0.02063
O	4.52141	0.33522	−0.10835
H	4.87100	1.23585	−0.13953

Frequencies (cm^{-1}): 37.11 65.96 94.91 131.50 204.86 221.13 314.95 355.84 371.05 415.05 415.90 512.96 515.85 544.64 562.76 628.67 654.46 690.97 746.85 807.64 815.72 844.03 871.97 890.56 917.74 946.17 972.61 1020.04 1024.52 1129.76 1176.31 1186.06 1199.54 1240.01 1295.83 1302.67 1329.86 1358.42 1371.51 1384.54 1473.96 1549.74 1624.69 1655.33 1681.91 1776.25 3170.77 3178.77 3192.61 3199.48 3215.15 3219.20 3774.14 3823.14

E = −573.490278890 hartrees

4-Hydroxy-Cinnamic Acid, Protonated

Atom	X	Y	Z
C	1.15712	−0.90505	−0.00367
C	2.53018	−0.85489	−0.04951
C	3.18419	0.39830	−0.11959
C	2.43963	1.59600	−0.14341
C	1.06186	1.53464	−0.09733
C	0.37626	0.28894	−0.02762
H	0.67195	−1.87344	0.05146
H	2.94830	2.55435	−0.19678
H	0.48710	2.45613	−0.11524
C	−1.04058	0.30036	0.01444
H	−1.50868	1.28279	−0.00189
C	−1.89133	−0.79562	0.07637
H	−1.53372	−1.81750	0.08178
C	−3.27471	−0.64821	0.13360
O	−3.82909	0.55154	0.22205
H	−4.78483	0.57632	0.05490
O	−4.01893	−1.74032	0.10778
H	−4.95931	−1.61018	0.31134
H	3.13436	−1.75512	−0.03219
O	4.52072	0.36916	−0.16059
H	4.90762	1.25687	−0.20703

Frequencies (cm^{-1}): 55.51 59.19 94.86 145.54 201.56 223.61 272.10 315.15 365.55 410.94 422.80 424.26 490.01 516.22 523.55 540.74 618.04 645.09 682.22 722.05 816.04 820.43 854.04 868.26 872.34 966.65 984.51 987.49 1010.53 1011.90 1134.07 1137.29 1157.60 1186.28 1205.96 1249.17 1310.45 1330.43 1356.21 1361.01 1409.04 1477.54 1497.57 1540.61 1591.53 1610.43 1626.90 1680.08 3181.58 3192.93 3207.08 3212.89 3230.26 3241.59 3771.75 3782.08 3789.74

E = −573.833425662 hartrees

4-Hydroxy-Ethyl Cinnamate

Atom	X	Y	Z
C	−1.84405	−1.38340	−0.00004
C	−3.22653	−1.49990	−0.00006
C	−4.02465	−0.34627	−0.00005
C	−3.42478	0.91712	−0.00004
C	−2.03493	1.01900	−0.00002
C	−1.21114	−0.12140	0.00000
H	−1.24608	−2.28902	−0.00003
H	−4.03860	1.81508	−0.00005
H	−1.57699	2.00461	−0.00001
C	0.24019	0.05556	0.00003
H	0.58347	1.08792	0.00009
C	1.18360	−0.90916	−0.00002
H	0.93851	−1.96607	−0.00009
C	2.63467	−0.65593	0.00002
O	2.96088	0.66182	0.00007
O	3.47111	−1.54559	−0.00001
H	−3.70854	−2.47192	−0.00009
O	−5.37933	−0.52409	−0.00011
H	−5.82891	0.33148	−0.00023
C	4.37480	0.96865	0.00011
H	4.83280	0.51309	−0.88343
H	4.83275	0.51308	0.88367
C	4.51727	2.47899	0.00012
H	4.05233	2.91844	0.88807
H	5.57881	2.74819	0.00015
H	4.05238	2.91845	−0.88786

Frequencies (cm^{-1}): 25.39 52.39 62.19 62.91 98.71 147.27 175.75 200.38 241.21 261.77 320.64 357.85 372.37 374.31 416.54 419.05 505.40 519.15 550.66 653.75 691.39 701.17 745.80 808.79 810.45 815.17 843.83 865.49 880.50 893.35 944.93 971.94 975.08 1016.09 1024.96 1073.13 1130.04 1134.64 1178.36 1185.60 1197.94 1229.93 1282.78 1292.91 1294.56 1304.74 1330.36 1361.08 1378.71 1399.95 1431.67 1473.32 1491.33 1503.21 1522.26 1549.85 1625.70 1655.91 1682.42 1756.27 3047.51 3063.88 3102.73 3119.25 3130.09 3169.82 3178.60 3190.88 3197.59 3210.54 3216.86 3824.55

E = −652.115973680 hartrees

4-Methyl-Cinnamic Acid

Atom	X	Y	Z
C	1.09009	−0.90768	−0.04939
C	2.47745	−0.83821	−0.05246
C	3.14966	0.39706	0.00471
C	2.37427	1.56142	0.06525
C	0.98060	1.49713	0.06860
C	0.30736	0.26412	0.01151
H	0.61027	−1.88022	−0.09464
H	2.86466	2.53034	0.11033
H	0.40211	2.41622	0.11624
C	−1.15568	0.25862	0.01810
H	−1.62474	1.23896	0.06809
C	−1.96985	−0.81671	−0.03060
H	−1.59736	−1.83397	−0.08199
C	−3.43602	−0.73500	−0.01982
O	−3.92883	0.53549	0.04450
H	−4.89631	0.45202	0.04492
O	−4.17569	−1.70396	−0.06369
H	3.05559	−1.75796	−0.10004
C	4.65812	0.45233	−0.00001
H	5.06808	−0.00334	−0.90899
H	5.02073	1.48257	0.05187
H	5.07708	−0.09531	0.85232

Frequencies (cm^{-1}): 24.96 37.12 65.13 94.60 129.27 195.13 220.25 301.21 352.20 371.95 416.48 503.52 511.02 541.71 565.58 626.65 655.03 701.72 747.35 779.50 830.39 845.49 866.68 900.05 917.99 965.17 981.00 1009.96 1022.92 1033.45 1061.66 1143.97 1178.27 1210.71 1235.22 1242.94 1295.41 1331.82 1347.42 1358.77 1378.58 1420.98 1449.29 1489.94 1498.23 1550.06 1606.94 1656.83 1683.26 1777.22 3036.40 3090.66 3122.89 3175.20 3175.66 3180.49 3192.29 3201.76 3217.94 3773.66

E = −537.584976879 hartrees

4-Methyl-Cinnamic Acid, Protonated

Atom	X	Y	Z
C	1.13369	−0.89095	−0.03612
C	2.51162	−0.81405	−0.05547
C	3.17700	0.43539	−0.03856
C	2.40218	1.60971	−0.00049
C	1.01825	1.54486	0.01869
C	0.34689	0.29383	0.00012
H	0.65777	−1.86579	−0.04800
H	2.89658	2.57588	0.01464
H	0.43537	2.46128	0.04848
C	−1.07653	0.29264	0.02139
H	−1.55033	1.27190	0.05877
C	−1.91539	−0.80910	−0.00049
H	−1.54869	−1.82664	−0.04876
C	−3.30492	−0.67652	0.03625
O	−3.86890	0.51287	0.15517
H	−4.83281	0.53198	0.04365
O	−4.03113	−1.77493	−0.04374
H	−4.98730	−1.66116	0.08131
H	3.09964	−1.72675	−0.08251
C	4.67703	0.49105	−0.06635
H	5.05894	0.06329	−1.00137
H	5.04903	1.51387	0.01814
H	5.10316	−0.10258	0.75037

Frequencies (cm^{-1}): 30.85 45.42 57.50 93.89 120.15 171.87 222.62 252.89 302.16 354.24 372.41 417.81 433.02 501.27 520.04 540.07 617.05 645.24 684.54 716.50 782.78 829.62 837.73 871.55 878.25 981.70 985.59 996.07 1010.55 1018.46 1025.77 1054.66 1127.46 1153.04 1160.44 1214.37 1240.95 1257.71 1311.38 1330.45 1351.54 1380.57 1416.93 1464.58 1480.91 1484.87 1493.16 1535.06 1576.12 1607.98 1626.60 1669.94 3042.96 3098.91 3147.45 3181.17 3194.39 3197.42 3211.17 3214.40 3241.79 3771.70 3783.32

E = −537.925185453 hartrees

4-Methyl-Ethyl Cinnamate

Atom	X	Y	Z
C	−1.82224	−1.37241	0.00029
C	−3.20793	−1.47459	0.00030
C	−4.02740	−0.33052	0.00012
C	−3.40097	0.92152	−0.00014
C	−2.00976	1.02936	−0.00015
C	−1.18915	−0.11212	0.00008
H	−1.22646	−2.27979	0.00042
H	−4.00671	1.82403	−0.00035
H	−1.54880	2.01393	−0.00037
C	0.26474	0.06330	0.00007
H	0.60957	1.09507	−0.00016
C	1.20408	−0.90425	0.00032
H	0.95486	−1.96017	0.00058
C	2.65733	−0.65647	0.00034
O	2.98827	0.65922	−0.00004
O	3.48909	−1.55023	0.00072
H	−3.66828	−2.45982	0.00043
C	−5.53166	−0.46052	0.00049
H	−5.88222	−1.00653	0.88423
H	−6.01713	0.51927	−0.00278
H	−5.88223	−1.01255	−0.87948
C	4.40337	0.96190	−0.00008
H	4.85997	0.50368	−0.88293
H	4.85947	0.50599	0.88425
C	4.55018	2.47179	−0.00196
H	4.08619	2.91370	0.88521
H	5.61250	2.73780	−0.00190
H	4.08687	2.91140	−0.89063

Frequencies (cm^{-1}): 24.93 33.39 52.56 61.22 62.93 96.81 146.29 171.68 192.16 241.63 261.31 304.59 349.70 362.18 389.07 416.57 502.91 506.52 546.72 654.48 698.56 699.80 745.56 782.15 810.44 829.88 844.90 861.46 879.41 899.72 963.72 975.01 979.94 1008.26 1018.95 1033.67 1061.17 1072.72 1134.42 1144.18 1178.27 1209.06 1232.24 1236.82 1283.54 1294.60 1296.19 1331.41 1347.22 1364.16 1399.79 1420.36 1431.63 1448.87 1490.19 1491.28 1498.17 1503.16 1522.10 1550.42 1607.43 1657.32 1683.72 1757.24 3035.93 3047.89 3064.18 3089.91 3103.19 3119.63 3122.06 3130.62 3173.48 3174.28 3180.22 3190.61 3199.68 3212.65

E = −616.210794243 hartrees

3. Potential Energy Curves, Obtained at the M06-2X/6-31+G(d,p) Level

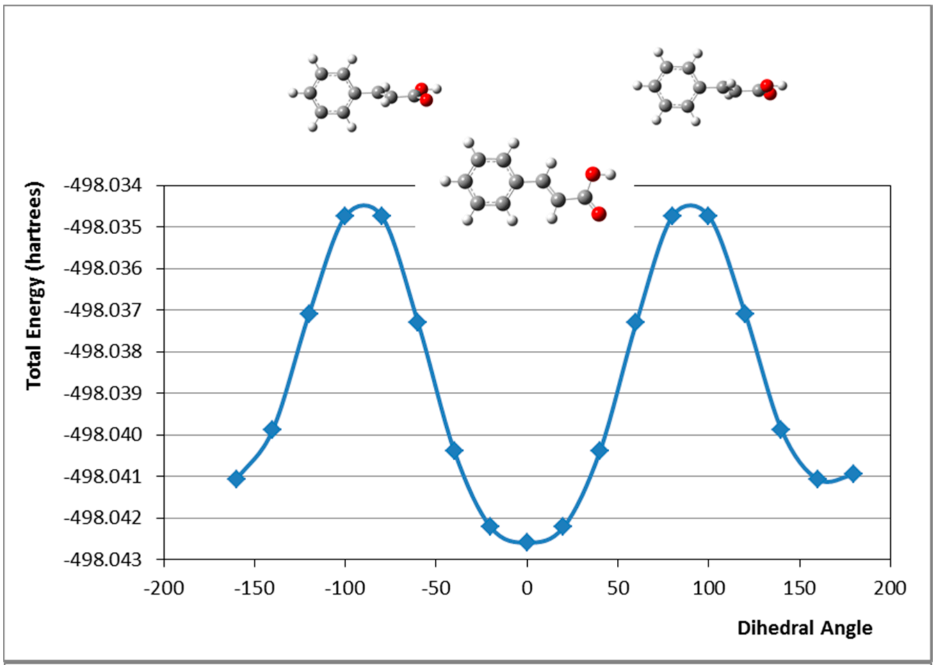

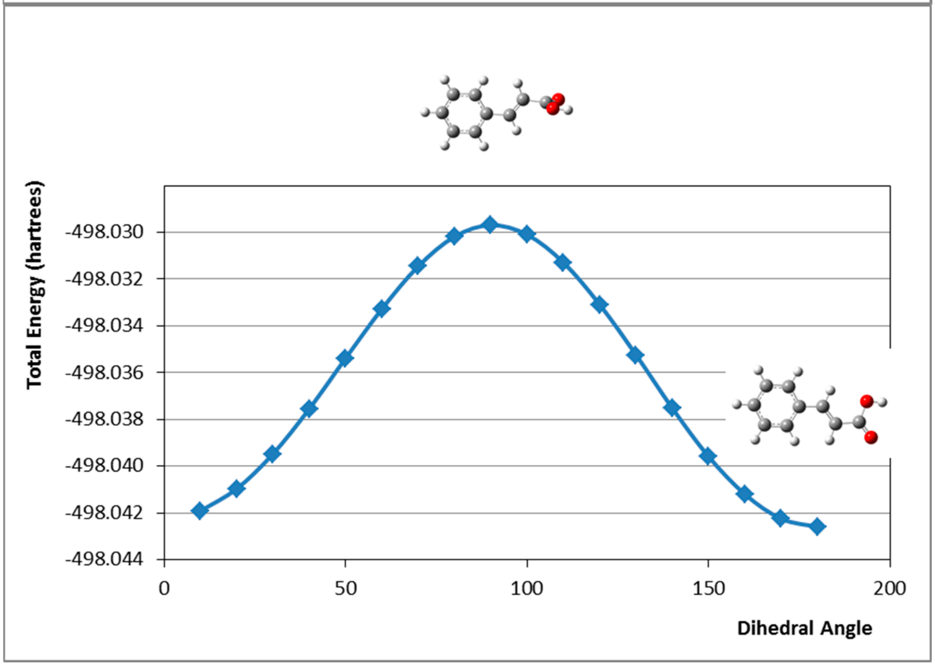

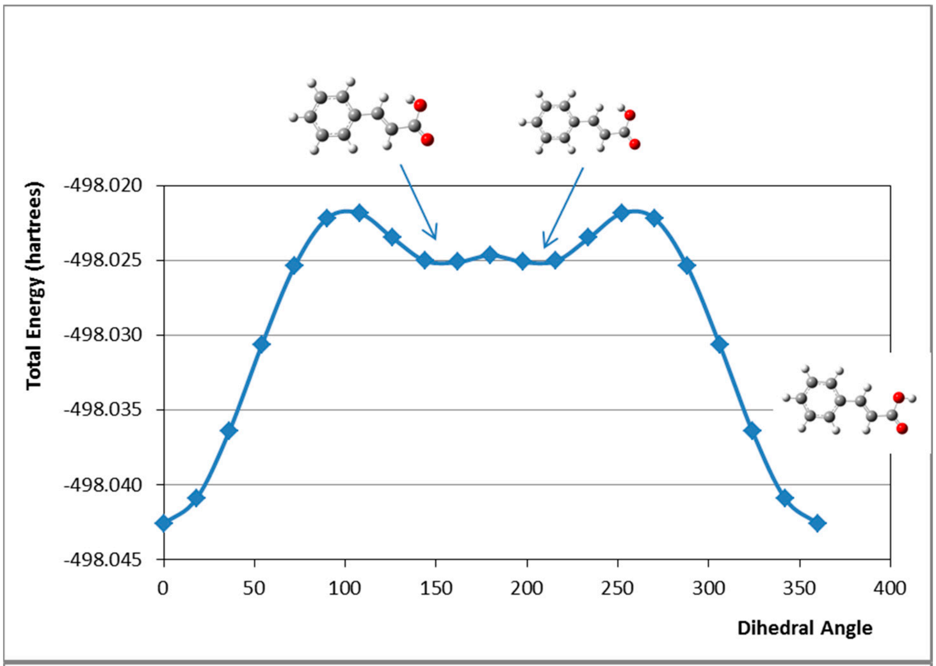
Total Energy (hartrees)
-498.020
-498.025
-498.030
-498.035
-498.040
-498.045
0 50 100 150 200 250 300 350 400
Dihedral Angle

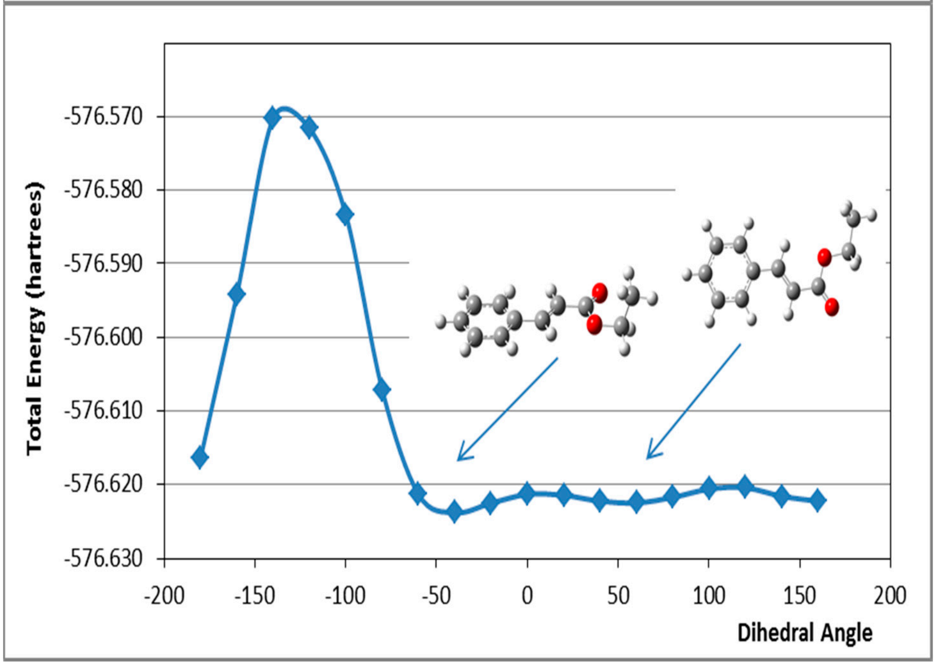
Total Energy (hartrees)
-576.570
-576.580
-576.590
-576.600
-576.610
-576.620
-576.630
-200 -150 -100 -50 0 50 100 150 200
Dihedral Angle
